# Immunoglobulin heavy-chain status and stromal interactions shape ferroptosis sensitivity in chronic lymphocytic leukemia

**DOI:** 10.1038/s41392-025-02535-x

**Published:** 2026-01-05

**Authors:** Martin Böttcher, Lea Reemts, Paul J. Hengeveld, Romy Böttcher-Loschinski, Vikas Bhuria, Junyan Lu, Silvia Materna-Reichelt, Durdam Das, Natasa Stojanović Gužvić, Heiko Bruns, Wolfgang Huber, Thorsten Zenz, Denny Schanze, Martin Zenker, Sascha Dietrich, Anton W. Langerak, Dimitrios Mougiakakos

**Affiliations:** 1 Department for Hematology, Oncology and Cell Therapy, Otto-von-Guericke University, Magdeburg, Germany; 2 Healthcampus Immunology, Inflammation and Infectiology (GC-I3), Otto-von-Guericke-University, Magdeburg, Germany; 3 Department of Immunology, Laboratory of Medical Immunology, Erasmus MC, University Medical Centre Rotterdam, Rotterdam, The Netherlands; 4 Genome Biology Unit, European Molecular Biology Laboratory, Heidelberg, Germany; 5 Medical Faculty Heidelberg, Heidelberg University, Heidelberg, Germany; 6 Fraunhofer Institute for Toxicology and Experimental Medicine ITEM-R, Regensburg, Germany; 7 Department of Medicine 5, Hematology and Oncology, Friedrich-Alexander-Universität Erlangen-Nürnberg and University Hospital Erlangen, Erlangen, Germany; 8 Department of Medical Oncology and Hematology, University Hospital Zurich, Zurich, Switzerland; 9 Institute of Human Genetics, University Hospital Magdeburg, Medical Faculty, Otto-von-Guericke University, Magdeburg, Germany; 10 Department of Hematology, Oncology and Clinical Immunology, Medical Faculty and University Hospital Düsseldorf, Heinrich-Heine-University Düsseldorf, Düsseldorf, Germany

**Keywords:** Haematological cancer, Haematological cancer

## Abstract

Chronic lymphocytic leukemia (CLL) is characterized by the accumulation of clonal B cells. Although targeted therapies have improved outcomes, resistance remains a challenge, particularly in high-risk patients with TP53 mutations or unmutated immunoglobulin heavy-chain variable region (IGHV) genes (U-CLL). Ferroptosis, a regulated, iron-dependent form of cell death, may represent an exploitable vulnerability in CLL; however, its mechanisms and clinical relevance remain poorly understood. Here, we identified IGHV status and microenvironmental cues as determinants of ferroptosis sensitivity. Using CLL cell lines, patient samples, and in vivo models, we show that CLL cells exhibit elevated basal levels of lipid peroxides and labile iron, predisposing them to ferroptosis. However, stromal interactions enhance cystine import and glutathione synthesis, thereby mitigating susceptibility to ferroptosis. Mechanistically, BTK inhibition sensitizes CLL cells to ferroptosis by increasing the transferrin receptor (TFRC, CD71) and increasing the intracellular Fe²⁺ level. High TFRC expression was associated with improved survival in two independent CLL patient cohorts, supporting its therapeutic and prognostic relevance. Combining ibrutinib with the GPX4 inhibitor RSL3 enhances ferroptosis and improves antileukemic efficacy in vivo. CLL cells with mutated IGHV genes (M-CLL) display greater TFRC expression and ferroptosis sensitivity than U-CLL cells do. This resistance can be overcome by ibrutinib-mediated TFRC induction or via metabolic targeting of fatty acid metabolism. Notably, ACSL1 is selectively upregulated in U-CLL cells and represents a targetable metabolic enhancer of ferroptosis sensitivity, as shown in vivo. Our findings reveal that TFRC and ACSL1 are functionally distinct yet targetable nodes that govern ferroptosis vulnerability in CLL patients and may guide novel therapeutic strategies for high-risk patients.

## Introduction

Chronic lymphocytic leukemia (CLL) is the most common leukemia in adults and is characterized by the progressive accumulation of clonal B cells in the bone marrow, lymph nodes, and peripheral blood.^1^ These are mature lymphocytes, the majority of which are quiescent, so their accumulation is not primarily due to increased proliferation but to defects in the apoptotic apparatus.^2^ Over the past decade, therapeutic innovations have fundamentally transformed CLL management, enabling largely chemotherapy-free treatment approaches through the introduction of BTK inhibitors (e.g., ibrutinib) and BCL2 inhibitors (e.g., venetoclax). These targeted agents exploit two central survival dependencies of CLL cells: chronic B-cell receptor (BCR) signaling mediated through BTK and a marked anti-apoptotic reliance on BCL2, respectively.^3,4^ Despite these advances, disease relapse and therapeutic resistance remain major challenges, particularly in patients with high-risk genetic features such as *TP53* mutations and unmutated immunoglobulin heavy-chain variable region (IGHV) genes.^5^ Moreover, accumulating evidence indicates that microenvironmental cues and metabolic adaptations profoundly influence CLL cell survival, shaping differential sensitivities to stress-induced cell death.^6–8^ These context-dependent vulnerabilities underscore the importance of investigating non-apoptotic pathways, such as ferroptosis, to identify novel therapeutic strategies.

Ferroptosis is an iron-dependent form of regulated cell death driven by the accumulation of lipid peroxides and characterized by distinct molecular mechanisms involving iron and lipid metabolism.^9^ Unlike apoptosis, ferroptosis is not regulated by caspases, making it an attractive target in apoptosis-resistant cancers, including hematologic malignancies.^10^ Ferroptosis is characterized by ferrous iron (Fe^2+^)-catalyzed lipid peroxidation and oxidative damage to cell membranes, which impairs their integrity. The Fenton reaction plays a central role in this process, where ferrous iron (Fe²⁺) reacts with hydrogen peroxide (H₂O₂), generating highly reactive hydroxyl radicals (•OH) while simultaneously producing ferric iron (Fe³⁺). The key regulators that promote ferroptosis include transferrin receptor (TFRC, CD71), ferritin, and acyl-CoA synthetase long-chain family members, which control iron uptake, storage, and lipid metabolism, influencing susceptibility to ferroptosis. Ferroptosis is counteracted by glutathione peroxidase 4 (GPX4), which reduces lipid peroxides to non-toxic lipid alcohols via the use of glutathione (GSH) as a cofactor, thereby maintaining membrane integrity. Additionally, the xCt cystine/glutamate antiporter plays a crucial role by importing cystine, which is converted into cysteine and subsequently used for GSH synthesis, supporting antioxidant defense. Ferritin, which is composed of ferritin heavy chain 1 (FTH1) and ferritin light chain (FTL), functions as a key iron storage protein complex, sequestering excess intracellular iron to prevent toxic iron accumulation and limit oxidative damage. FTH1 possesses ferroxidase activity, converting ferrous iron (Fe²⁺) to ferric iron (Fe³⁺) for safe storage, whereas FTL contributes to the stability and assembly of the ferritin complex. By regulating intracellular iron levels, ferritin plays a critical role in counteracting ferroptosis through limiting Fe²⁺ availability.

While ferroptosis has been implicated in aggressive lymphomas,^11,12^ its role in CLL remains largely unexplored. Given the inherent oxidative stress observed in CLL cells^6^ and their dependence on lipid metabolism,^13^ ferroptosis may represent a novel therapeutic vulnerability. In addition to cell intrinsic features, the stromal microenvironment within the bone marrow and lymph node niches plays a pivotal role in CLL pathogenesis and therapy resistance.^14^ Through cytokine signaling, direct cell-cell interactions, and metabolic crosstalk, CLL cells establish a protective niche that enhances their survival by, among other things, adapting their metabolism, increasing their antioxidant capacity, and mitigating oxidative stress.^7,15^ However, whether stromal cells actively modulate iron homeostasis and glutathione metabolism to confer resistance to ferroptosis in CLL requires further investigation. A recent study suggested that BTK and BCL2 inhibitors may modulate ferroptosis susceptibility in patients with lymphoid malignancies.^16^ While ibrutinib has broad effects on CLL cell signaling^17^ and metabolism,^18^ its effect on ferroptosis induction is unknown. Venetoclax, a potent BCL2 antagonist, is highly effective in treating CLL. Hence, whether these agents induce ferroptosis as part of their mechanism of action or sensitize CLL cells to ferroptotic cell death remains to be elucidated.

In this study, we aimed to define the ferroptosis susceptibility of CLL cells, investigate how the stromal microenvironment influences ferroptosis resistance, and evaluate whether targeted therapies such as BTK and BCL2 inhibitors modulate ferroptosis-induced cell death. In addition, we investigated the impact of the mutational status of CLL cells on ferroptosis susceptibility and identified potential metabolic vulnerabilities that could enhance ferroptotic cell death. We found that ferroptosis susceptibility is not uniform across CLL but is shaped by IGHV status and microenvironmental cues, converging on TFRC-dependent iron acquisition and long-chain acyl-CoA synthetase 1 (ACSL1)–mediated lipid metabolism as key regulators. These pathways emerged as targetable determinants of ferroptotic cell death. By revealing novel mechanisms that regulate ferroptosis in CLL, our findings establish ferroptosis as a potential therapeutic vulnerability and highlight metabolic dependencies that could be exploited to overcome therapy resistance and improve patient outcomes, as supported by our preclinical in vitro and ex vivo models, confirming that pharmacologic ferroptosis induction is feasible in CLL.

## Results

### CLL cells are sensitive to ferroptosis

**Fig. 1:**
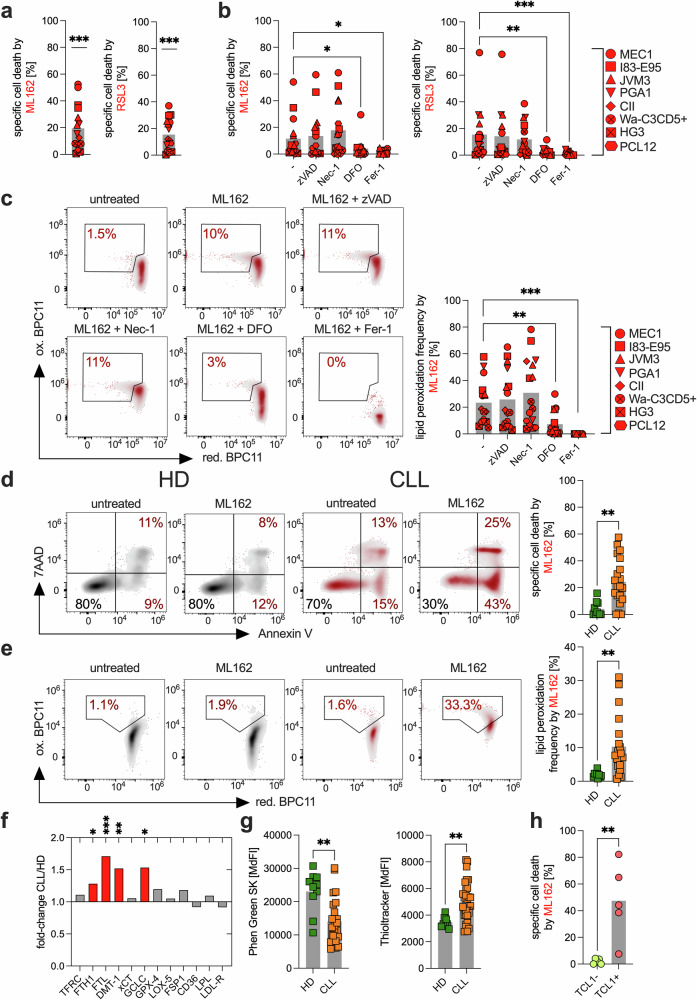
CLL cells are sensitive to ferroptosis induction. **a** The CLL cell lines CII, HG3, I83-E95, JVM-3, Mec-1, PCL-12, PGA-1, and Wa-C3CD5+ were treated in two independent experiments with the GPX4 inhibitor ML162 (100 nM) or RSL3 (100 nM) for 4 h, and compound-triggered specific cell death was determined. Specific cell death was calculated relative to the untreated control as follows: 100 × (% dead cells − % baseline)/(100 − % baseline). The baseline values were normalized to 0%. **b** The same set of CLL cell lines was subjected to two independent experiments with the GPX4 inhibitors ML162 or RSL3 in the presence/absence of zVAD (apoptosis inhibitor, 10 µM), necrostatin-1/Nec-1 (necroptosis inhibitor, 100 µM), and deferoxamine (DFO, 100 µM) or ferrostatin-1/Fer-1 (ferroptosis inhibitors, 25 µM). **c** In the same samples, lipid peroxidation was assessed by flow cytometry (FACS) using BODIPY™ 581/591 C11, as shown in the representative analysis in the left panel. The data are summarized in the right panel. **d** Specific cell death of B cells from healthy donors (HD, n = 10) and CLL cells from patients (CLL, n = 20) was assessed via FACS following 24 h of ML162 treatment (500 nM), as shown in the representative analysis in the left panel. The data are summarized in the right panel. **e** Lipid peroxidation of B cells from healthy donors (HD, n = 10) and CLL cells from patients (CLL, n = 20) was assessed via FACS following 24 h of ML162 treatment (500 nM), as shown in the representative analysis in the left panel. The data are summarized in the right panel. **f** Expression of key pro- and anti-ferroptotic proteins was analyzed in primary CLL cells (CLL, n = 31) and HD B cells (HD, n = 12) on the basis of the median fluorescence intensity (MdFI) directly ex vivo. The data are shown as the fold change between CLL/HD patients. Statistical significance was determined on the basis of groupwise comparisons of MdFI values. Significantly differentially expressed proteins are highlighted in red. **g** Intracellular ferrous iron (Fe^2+^) (HD, n = 10 and CLL, n = 24, left panel) and glutathione (HD, n = 8 and CLL, n = 34, right panel) baseline levels were measured via FACS via Phen Green SK and Thioltracker™, respectively. Note that the Phen Green SK signal is quenched by Fe²⁺; thus, lower fluorescence indicates higher intracellular ferrous iron levels. **h** Specific cell death was assessed in B cells and CLL cells from nontransgenic littermates (TCL1-, n = 5) and transgenic Eµ-TCL1 mice (TCL1+, n = 5) following ex vivo treatment for 24 h with 500 nM ML162. Statistical analysis: paired t-tests were applied for comparisons involving dependent (matched) samples (**a**), whereas unpaired t-tests were used for comparisons between independent groups (**d**–**h**). One-way ANOVA with multiple comparisons was used to assess differences across multiple treatment conditions (**b**, **c**). ‘n’ indicates the sample number, bars represent the mean; P-value: *P < 0.05; **P < 0.01; ***P < 0.001

To investigate whether CLL cells respond to ferroptosis-inducing agents, eight different CLL cell lines (i.e., HG3, CII, PCL-12, Wa-C3CD5+, Mec-1, PGA-1, I83-E95, and JVM-3) were treated with GPX4 inhibitors (i.e., ML162 and RSL3) and an inhibitor of the xCt cystine transporter (i.e., erastin). Treatment with ML162 and RSL3 induced cell death, which could be prevented by ferroptosis inhibitors such as deferoxamine (DFO) and ferrostatin-1 (Fer-1) but not by apoptosis or necroptosis inhibitors (Fig. 1a, b). Despite statistical significance, we noted interline variability in response, likely reflecting the biological heterogeneity of CLL cell lines. Consistently, ML162 and RSL3 increased lipid peroxidation in the CLL cell lines, which was reversed upon treatment with ferroptosis inhibitors (Fig. 1c). Additionally, ML162 and RSL3 led to a decrease in intracellular ferrous iron (Fe^2^^+^), likely due to enhanced oxidation during the Fenton reaction,^19^ converting Fe^2+^ to Fe^3+^ (Supplementary Fig. 1a, b). Moreover, we did not observe any effect of ML162 or RSL3 on intracellular ROS levels, mitochondrial ROS levels, or intracellular thiol levels (Supplementary Fig. 1c–e). In contrast, erastin failed to induce ferroptosis in our in vitro model, potentially because, as previously published, CLL cells are inherently limited in their ability to take up cystine under baseline conditions (Supplementary Fig. 1f–i).^15^

Next, we examined whether ferroptosis could be induced in primary CLL cells and whether their sensitivity differed from that of healthy B cells. Indeed, primary CLL cells were significantly more sensitive to GPX4 inhibition than healthy B cells were (Fig. 1d, e). In our previous studies, we demonstrated that CLL cells exhibit increased intracellular ROS levels,^6^ which may contribute to increased lipid peroxidation. In line with these findings, the baseline rate of lipid peroxidation in CLL cells was significantly greater (Supplementary Fig. 1j).

When we examined key pro- and anti-ferroptotic molecules at the protein level, we detected significantly increased expression of ferritin heavy chain 1 (FTH1), ferritin light chain 1 (FTL1), divalent metal transporter 1 (DMT1), and glutamate-cysteine ligase catalytic subunit (GCLC) in primary CLL cells compared with healthy B cells (Fig. 1f, Supplementary Fig. 1k). While FTH1, FTL1, and DMT1 are involved in iron metabolism, GCLC is the key enzyme in glutathione (GSH) synthesis. Consistent with these findings, both the intracellular Fe^2^^+^ level (pro-ferroptotic) and the glutathione level (anti-ferroptotic) were elevated in CLL cells (Fig. 1g). The increased Fe^2+^ levels may explain the increased sensitivity of CLL cells to ferroptosis, whereas elevated GSH levels may represent an intrinsic compensatory protective mechanism.

Finally, we validated our findings in a preclinical CLL model (i.e., Eµ-TCL1 transgenic mice), which recapitulates key features of aggressive CLL and is widely considered to reflect the biology of CLL with unmutated IGHV (U-CLL).^20^ This finding is supported by the observation that TCL1 expression is significantly higher in U-CLL cells than in their counterparts with mutated IGHV (M-CLL).^21^ Similarly, the malignant cells from these mice were significantly more sensitive to ferroptosis than were the nonmalignant B cells isolated from their nontransgenic littermates (Fig. 1h, Supplementary Fig. 1l).

### Stromal cells increase CLL cell resilience toward ferroptosis

**Fig. 2:**
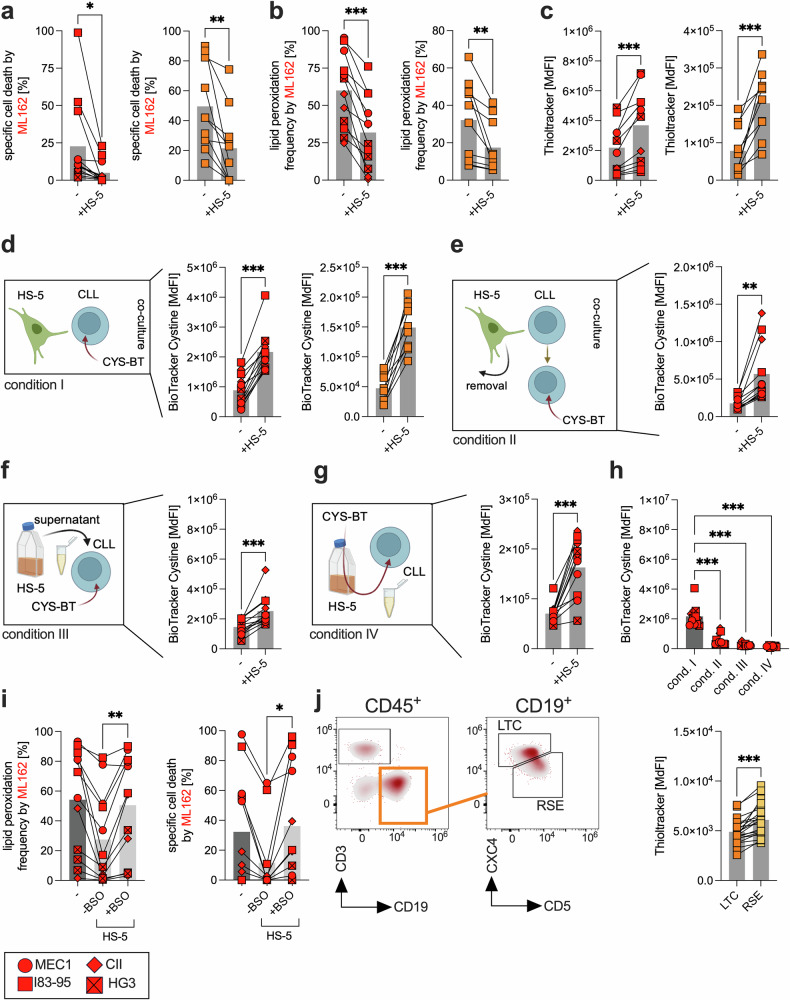
Stromal cells increase CLL cell resilience to ferroptosis. The CLL cell lines CII, HG3, I83-E95, and Mec-1 (three independent experiments) and primary CLL cells (orange squares, n = 10) were cultured in the presence/absence of HS-5 cells for 24 h and 48 h, respectively, followed by treatment with ML162 (100 nM), and **a** specific cell death and **b** lipid peroxidation were assessed by flow cytometry (FACS). Specific cell death was calculated relative to the untreated control as follows: 100 × (% dead cells − % baseline)/(100 − % baseline). The baseline values were normalized to 0%. **c** CLL cell lines CII, HG3, I83-E95, and Mec-1 (three independent experiments) and primary CLL cells (orange squares, n = 10) were cultured in the presence/absence of HS-5 cells for 24 h and 48 h, and the levels of intracellular thiols (i.e., glutathione) were assessed via FACS. Uptake of FITC-conjugated cystine (i.e., BioTracker^TM^ cystine, CYS-BT) by CLL cells was evaluated under different conditions. First, **d** CII, HG3, I83-E95, Mec-1 (three independent experiments), and primary CLL cells (orange squares, n = 10) were cultured in the presence or absence of HS-5 cells for 24 h and 48 h, respectively. CYS-BT was added to the culture 30 min prior to measurement (=condition I). **e** CYS-BT was applied to the CLL cell lines following their coculture with and separation from HS-5 cells (=condition II). **f** CLL cell lines were cultured in the presence or absence of HS-5-derived CM, and CYS-BT was added to the medium (=condition III). **g** HS-5 cells were cultured in the presence of CYS-BT. The medium (including metabolized and secreted CYS-BT as well as any CYS-BT that was not taken up by HS-5 cells) was then collected and subsequently added to the culture of CLL cell lines (=condition IV). **h** Uptake of CYS-BT quantified on the basis of the CYS-BT median fluorescence intensity (MdFI) detected in CLL cells is summarized for all four conditions (cond.) I–IV. **i** CII, HG3, I83-E95, and Mec-1 (three independent experiments) were cocultured with/without HS-5 cells in the presence/absence of the inhibitor of GSH synthesis buthionine sulfoximine (BSO, 100 µM) for 24 h. Following treatment for 4 h with ML162 (100 nM), lipid peroxidation and specific cell death were assessed via FACS. **j** The left panel shows a representative FACS-based gating strategy for the CLL subpopulation of CD5^high^CXCR4^low^ recent stromal emigrant (RSE) and CD5^low^CXCR4^high^ long-term circulating (LTC) cells. Intracellular glutathione was semiquantified via ThiolTracker™ MdFI in RSE and LTC CLL cells from 30 patients, as shown in the right panel. Statistical analysis: Paired t-tests were applied for comparisons involving dependent (matched) samples (**a**–**g**, **j**), whereas one-way ANOVA with multiple comparisons was used to assess differences across multiple treatment conditions (**h**, **i**). ‘n’ indicates the sample number, bars represent the mean; P-value: *P < 0.05; **P < 0.01; ***P < 0.001

We and others have previously demonstrated that contact with stromal cells reduces CLL cell sensitivity to spontaneous apoptosis,^14^ therapeutics,^7^ and immunosurveillance.^8^ To explore this further, we investigated the effects of the human bone marrow stromal cell line HS-5 on CLL cells. Indeed, coculture of HS-5 cells with CLL cells (both cell lines and primary CLL cells) significantly reduced their sensitivity to GPX4 inhibition (Fig. 2a, b). Overall, the antioxidative potential of CLL cells was enhanced following coculture with HS-5, as evidenced by increased GPX4 expression (Supplementary Fig. 2a, b). Additionally, we observed elevated intracellular thiol levels, which largely reflect the GSH content (Fig. 2c). Consistent with these findings, we noted stromal contact-mediated induction of GCLC and xCt expression (Supplementary Fig. 2c, d). GCLC, a key enzyme in GSH synthesis, likely contributes to increased GSH production, whereas xCt can enhance the import of intracellular cystine, which is used for GSH synthesis. In support of a transcriptional mechanism, we further observed that stromal contact significantly upregulated the expression of nuclear factor erythroid 2–related factor 2 (NRF2), a master regulator of GPX4, GCLC, and xCT, in CLL cells (Supplementary Fig. 2e).^22^ Similar protective effects were observed when CLL cells were cocultured with the alternative bone marrow stromal cell line HS-27A, where ML162-induced specific cell death and lipid peroxidation were likewise reduced, accompanied by a significant increase in the concentration of intracellular thiols (Supplementary Fig. 2f–h). Similar phenotypes, including increased thiol content and increased ferroptosis resistance, were also observed when healthy donor-derived mesenchymal stromal cells (pMSCs) were cocultured with primary CLL cells, further underscoring the relevance of stroma-mediated redox modulation across model systems (Supplementary Fig. 2i, j).

Notably, cystine uptake was significantly increased in CLL cells in direct contact with stromal cells (Fig. 2d). Even after CLL cells were separated from stromal cells, the CLL cells retained an improved capacity for cystine uptake (Fig. 2e).

To further understand the crosstalk between CLL cells and stromal cells, we treated CLL cells with conditioned medium (CM) from HS-5 cells. This also resulted in significantly improved cystine uptake (Fig. 2f). Previous studies have shown that stromal cells take up cystine, convert it to cysteine, and release it into the microenvironment, where it can be taken up by CLL cells and used for GSH synthesis.^15^ To confirm this, we treated HS-5 cells with FITC-labeled cystine, collected their conditioned medium, and exposed CLL cells to it. We observed a significant signal increase in CLL cells, driven by unmetabolized cystine and/or its metabolite cysteine derived from stromal cells, which is consistent with previous reports (Fig. 2g).^15^ However, the most pronounced effects were observed under direct cell‒to‒cell contact conditions (Fig. 2h).

The critical role of GSH synthesis in this process was highlighted by the fact that the protective effects of the stromal interaction were nearly completely abrogated by treatment with buthionine sulfoximine (BSO), which interferes with GSH synthesis, or sorafenib, which blocks cystine uptake (Fig. 2i, Supplementary Fig. 2k).

Research on compartmental trafficking indicates that CLL cells actively proliferating within the bone marrow or lymph node niches exhibit upregulation of CD5 and downregulation of CXCR4, leading to the formation of the CD5^high^CXCR4^low^ subset, referred to as recent stromal-niche emigrants (RSEs).^23^ Once these cells migrate into peripheral blood, they transition into a quiescent state, reduce their proliferative activity, downregulate CD5, and restore CXCR4 expression,^24^ resulting in the CD5^low^CXCR4^high^ subset, known as long-term circulating cells (LTCs). Consistent with our in vitro model of CLL-stroma interactions, we found that CLL patient-derived RSE subsets presented increased GPX4 and GCLC expression, along with increased GSH content. In contrast, xCt expression was higher in LTCs, although it remains unclear whether this represents a compensatory response to lower GSH levels. However, the capacity for cystine uptake remained greater in the RSE subset (Fig. 2j, Supplementary Fig. 2l-o).

### Bruton’s tyrosine kinase (BTK) and Bcl-2 inhibitors induce ferroptosis, but only BTK inhibition sensitizes CLL cells to ferroptosis

**Fig. 3:**
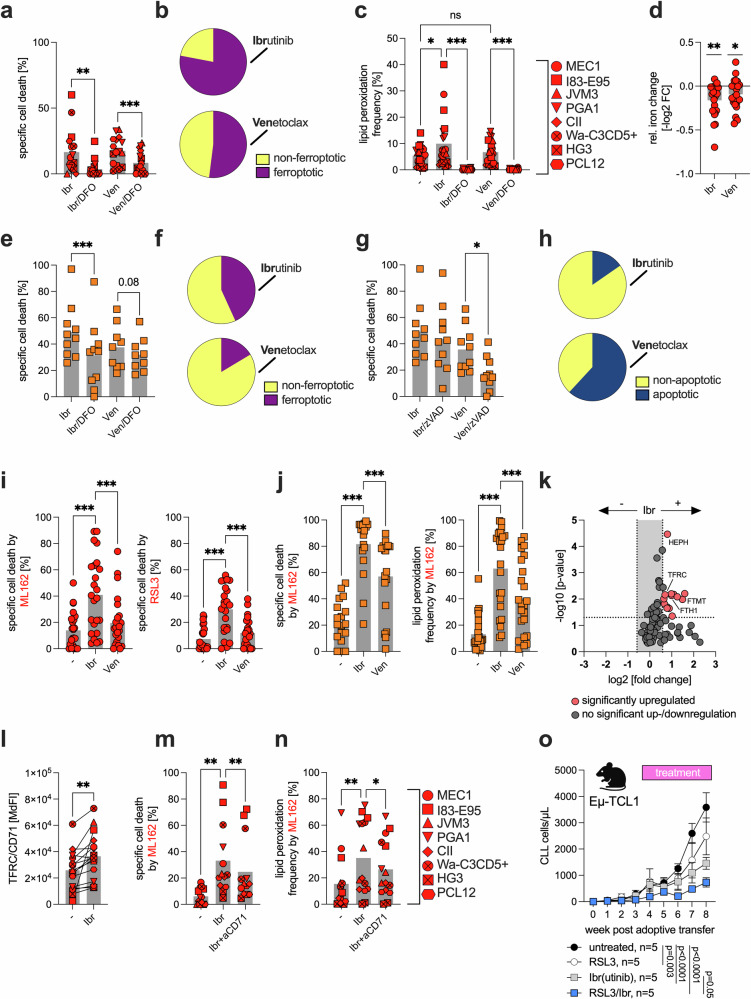
Impact of BTK and BCL2 inhibition on the induction of and/or sensitization to ferroptosis in CLL cells. **a** The CLL cell lines CII, HG3, I83-E95, JVM-3, Mec-1, PCL-12, PGA-1, and Wa-C3CD5+ were cultured in two independent experiments for 24 h in the presence or absence of ibrutinib (Ibr, 10 µM) or venetoclax (Ven, 50 nM), with or without deferoxamine (DFO, 100 µM). Cell viability was assessed by flow cytometry (FACS), and specific types of cell death were determined. Specific cell death was calculated relative to that of the control (=baseline) as follows: 100 × (% dead cells − % baseline)/(100 − % baseline). The baseline values were normalized to 0%. **b** The contribution of ferroptosis to the overall cytotoxicity induced by ibrutinib or venetoclax is shown. The calculation is based on the rescue potential of the DFO. In addition, **c** we assessed the accompanying changes in lipid peroxidation and **d** relative changes in intracellular ferrous iron (Fe^2+^) levels. **e** Primary CLL samples (n = 10, orange squares) were cultured for 24 h in the presence or absence of ibrutinib (Ibr, 10 µM) or venetoclax (Ven, 1 nM) with or without DFO (100 µM), and specific cell death and **f** the contribution of ferroptosis to overall cytotoxicity were assessed. **g** Patient-derived CLL samples (n = 10, orange squares) were cultured for 24 h in the presence or absence of ibrutinib (Ibr, 10 µM) or venetoclax (Ven, 1 nM) with or without zVAD (apoptosis inhibitor, 10 µM). Specific cell death was determined by FACS, and **h** the contribution of apoptosis to the overall cytotoxicity induced by ibrutinib or venetoclax is shown. The calculation is based on the rescue potential of zVAD. **i** CLL cell lines (red circles) pretreated for 24 h with/without ibrutinib (Ibr, 10 µM) or venetoclax (Ven, 50 nM) were subsequently treated with 100 nM ML162 or 100 nM RSL3, and specific cell death was assessed. **j** Primary CLL cells (orange squares) were pretreated for 24 h with/without ibrutinib (Ibr, 10 µM) or venetoclax (Ven, 1 nM) and subsequently treated with 500 nM ML162, and specific cell death (n = 18, left panel) and lipid peroxidation (n = 23, right panel) were assessed. **k** Relative gene expression of 88 ferroptosis-related genes was analyzed in the CLL cell lines CII, HG3, I83-E95, JVM-3, Mec-1, PCL-12, PGA-1, and Wa-C3CD5+, which were cultured for 24 h in the absence or presence of 10 µM ibrutinib, via a qPCR array. The volcano plot displays relative gene expression (log₂-fold change) versus −log₁₀ p-values, with red dots indicating genes whose expression was significantly upregulated (i.e., p-value ≤ 0.05 and at least 1.5-fold) upon ibrutinib treatment. **l** The CLL cell lines CII, HG3, I83-E95, JVM-3, Mec-1, PCL-12, PGA-1, and Wa-C3CD5+ were treated in two independent experiments for 24 h with or without 10 µM ibrutinib, and transferrin receptor (TFRC, CD71) surface protein was measured via FACS. **m** CLL cell lines CII, HG3, I83-E95, JVM-3, Mec-1, PCL-12, PGA-1, and Wa-C3CD5+ were treated in three independent experiments for 24 h with either an IgG isotype control (-) or an anti-CD71 blocking antibody (aCD71, 2 µg/mL) in the absence/presence of ibrutinib (Ibr, 10 µM), followed by GPX4 inhibition with 100 nM ML162 for 4 h. Specific cell death and **n** lipid peroxidation were assessed via FACS. **o** Splenocytes from transgenic Eµ-TCL1 mice (≥80% CLL cells) were adoptively transferred into wild-type recipients (n = 5 per group) via i.v. injection. Disease burden was monitored over an 8-week period by measuring CLL cell counts (cells/µL) via FACS. Treatments were initiated between weeks 3 and 4 (as indicated) and included ibrutinib (0.16 mg/mL in drinking water ad libitum), RSL3 (100 mg/kg i.p. twice weekly), or a combination of both. Statistical analysis: Paired t-tests were applied for comparisons involving dependent (matched) samples (**a**, **d**, **e**, **g**, **k**, **l**), whereas one-way ANOVA with multiple comparisons was used to assess differences across multiple treatment conditions (**c**, **i**, **j**, **m**, **n**). Two-way ANOVA was applied for analysis of experiments involving multiple factors, such as treatment conditions and time points (**o**). ‘n’ indicates the sample number, bars represent the mean, error bars represent the standard error of the mean; P-value: *P < 0.05; **P < 0.01; ***P < 0.001

Next, we aimed to investigate whether common targeted therapies for CLL, specifically those that inhibit BTK (e.g., ibrutinib) or BCL2 (e.g., venetoclax), exert effects through the induction of ferroptosis. To test this hypothesis, CLL cells were treated with ibrutinib or venetoclax, either alone or in combination with DFO, a ferroptosis inhibitor. Treatment with both ibrutinib and venetoclax induced significant cell death, which was significantly, although not completely, antagonized by DFO (Fig. 3a). Notably, the proportion of cell death attributable to ferroptosis was markedly greater for ibrutinib than for venetoclax (Fig. 3b). This process was accompanied by increased lipid peroxidation and depletion of intracellular Fe^2+^, similar to what we observed with GPX4 inhibition (Fig. 3c, d; Supplementary Fig. 3a, b). In patient-derived CLL cells, we confirmed this differential response (Fig. 3e, f). Moreover, co-treatment with the pan-caspase inhibitor zVAD, which serves as a functional control for apoptosis inhibition, significantly reduced venetoclax-induced cell death, indicating a substantial apoptotic contribution of a BCL2 inhibitor, as expected. In contrast, zVAD had a minimal effect on ibrutinib-induced cell death, suggesting that apoptosis plays only a minor role, whereas ferroptosis contributes more prominently to the cytotoxic effects of ibrutinib (Fig. 3g, h).

We observed a significant increase in intracellular and mitochondrial ROS following ibrutinib treatment, whereas the intracellular GSH levels remained unchanged (Supplementary Fig. 3c–e). Treatment of primary CLL cells with the antioxidant N-acetylcysteine (NAC), which replenishes intracellular GSH and scavenges ROS, significantly reduced ibrutinib-induced ferroptosis and lipid peroxidation (Supplementary Fig. 3f). When ibrutinib was combined with venetoclax, we did not observe an increase in the contribution of ferroptosis beyond that induced by ibrutinib alone (Supplementary Fig. 3g).

Next, we sought to determine whether ibrutinib and/or venetoclax could increase sensitivity to GPX4 inhibition. In CLL cell lines, the strongest effect was observed for ibrutinib, which we also confirmed in primary CLL cells (Fig. 3i, j). Notably, ibrutinib was also able to overcome the protective effect of stromal cells on ML162-induced ferroptosis, further underscoring its role as a ferroptosis sensitizer (Supplementary Fig. 3h).

As ferroptosis induction by ibrutinib appeared heterogeneous across primary CLL samples, we next investigated whether lipid peroxidation levels induced by ibrutinib alone or in combination with the GPX4 inhibitor ML162 were correlated with ferroptotic cell death. While ibrutinib-induced lipid peroxidation did not significantly correlate with the degree of specific cell death, a strong positive correlation was observed between ML162-induced lipid peroxidation and ferroptosis under co-treatment with ibrutinib (Supplementary Fig. 3i). These data suggest that the sensitizing effects of ibrutinib are likely more pronounced than its direct ferroptosis-inducing activity, a finding that may be particularly relevant for rational combination strategies aimed at exploiting ferroptosis in CLL.

To better understand the effects of ibrutinib on pro- and anti-ferroptotic signaling pathways and molecules, we conducted an expression analysis of ferroptosis-related genes in ibrutinib-pretreated versus untreated cell lines. Ibrutinib treatment led to increased expression of genes involved in iron metabolism, including hephaestin (HEPH), transferrin receptor (TFRC, also known as CD71), ferritin mitochondrial (FTMT), and ferritin heavy chain 1 (FTH1) (Fig. 3k).

We further validated the upregulation of TFRC/CD71 in CLL cells after in vitro and in vivo ibrutinib treatment (Fig. 3l, Supplementary Fig. 4a, b). Interestingly, co-treatment with the antioxidant NAC significantly attenuated ibrutinib-induced upregulation of TFRC/CD71, suggesting that oxidative stress may be mechanistically involved in this process (Supplementary Fig. 4c). One possible explanation is that elevated intracellular ROS activate iron regulatory proteins such as IRP1 and IRP2,^25^ which in turn increase the expression of iron metabolism–related genes, including TFRC/CD71, DMT1, and FTL.

Importantly, blocking TFRC/CD71 with specific antibodies significantly reduced ferroptosis induction by ML162, suggesting that the ibrutinib effect could be at least partially explained by its ability to modulate TFRC/CD71 (Supplementary Fig. 4d). To further substantiate this mechanistic link, we performed genetic ablation of TFRC/CD71 via CRISPR-Cas9 in four different CLL cell lines. This intervention significantly diminished ML162-induced cell death, lipid peroxidation, and labile Fe²⁺ accumulation, confirming the functional importance of CD71 in mediating ferroptosis sensitivity in CLL (Supplementary Fig. 4e–h). This finding was further supported by experiments showing that the sensitizing effect of ibrutinib on CLL cells toward ferroptosis could be antagonized by TFRC/CD71 blockade (Fig. 3m, n).

Finally, we evaluated the combination of ferroptosis induction and ibrutinib in vivo via adoptive transfer in a TCL1 mouse model of CLL. To induce ferroptosis, we employed the GPX4 inhibitor RSL3, for which substantial preclinical data already exist.^26^ In vivo, the combination of ibrutinib and RSL3 was superior to either monotherapy alone, both of which significantly delayed disease progression, further supporting our previous observations (Fig. 3o, Supplementary Fig. 4i).

### IGHV mutational status determines CLL cell sensitivity to ferroptosis

Among the most important genetic prognostic factors in CLL are the IGHV mutational status and mutations in the *TP53* gene. Mutations in *TP53* can inhibit ferroptosis,^27^ whereas an unmutated IGHV status (U-CLL) and a mutated *TP53* gene (*TP53* MT) are associated with poor prognosis. We grouped the CLL cell lines in our study according to their mutational status into the following categories: *TP53* MT, U-CLL (i.e., PCL-12, CII), *TP53* WT, U-CLL (i.e., HG3, Wa-C3CD5+), *TP53* MT, M-CLL (i.e., MEC-1), and *TP53* WT, M-CLL (i.e., PGA-1, JVM-3, I83-E95).

**Fig. 4:**
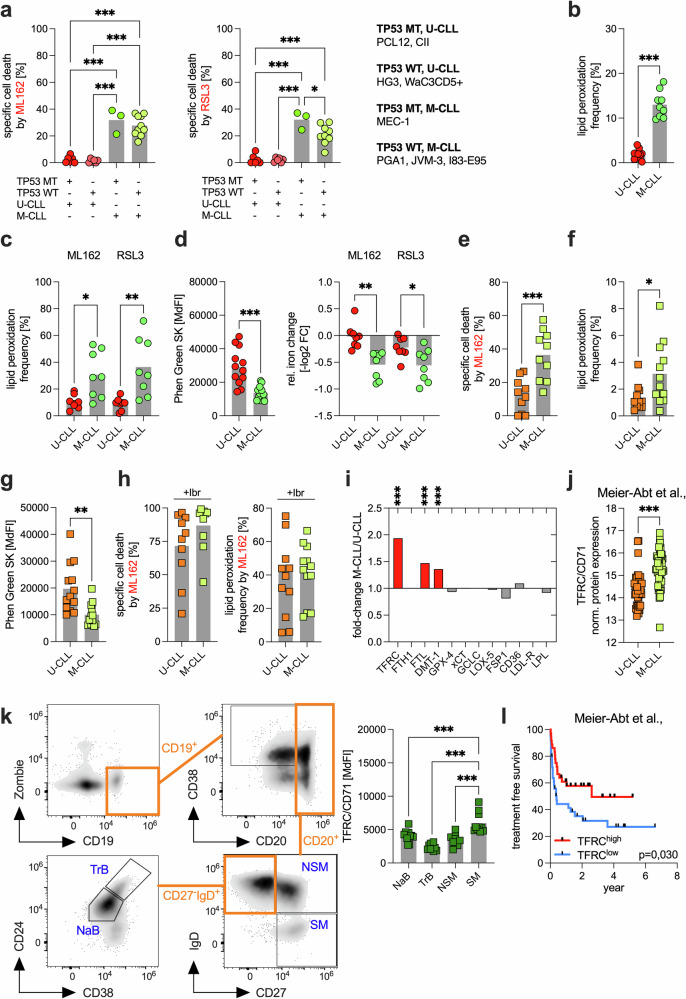
CLL cell sensitivity to ferroptosis is determined by the IGHV mutational status. **a** On the basis of a combination of their mutational status for *TP53* (mutated, MT, and wild-type, WT) and IGHV (unmutated, U-CLL, and mutated, M-CLL), the CLL cell lines were grouped accordingly into the following: *TP53* MT and U-CLL, *TP53* WT and U-CLL, *TP53* MT and M-CLL, and *TP53* WT and M-CLL. Ferroptosis was triggered by 100 nM ML162 or 100 nM RSL3, and specific cell death was assessed. Specific cell death was calculated relative to that of the control (=baseline) as follows: 100 × (% dead cells − % baseline)/(100 − % baseline). The baseline values were normalized to 0%. **b** Lipid peroxidation levels were measured in U-CLL (CII, HG3, PCL-12, and Wa-C3CD5+) and M-CLL (I83-E95, JVM-3, MEC-1, and PGA-1) cells under basal conditions in three independent experiments. **c** U-CLL and M-CLL cell lines were treated with 100 nM ML162 or 100 nM RSL3 in two independent experiments, and lipid peroxidation was assessed. **d** Baseline Fe²⁺ content (left panel, three independent experiments) and its relative change upon treatment with 100 nM ML162 and 100 nM RSL3 (right panel, two independent experiments) were assessed in U-CLL and M-CLL cell lines via FACS via Phen Green SK. Note that the Phen Green SK signal is quenched by Fe²⁺; thus, lower fluorescence indicates higher intracellular ferrous iron levels. **e** Primary patient U-CLL (n = 10) and M-CLL (n = 10) cells were treated with 500 nM ML162, and specific cell death was assessed. **f** Baseline lipid peroxidation is shown for primary patient U-CLL (n = 12) and M-CLL cells (n = 12). **g** Baseline Fe²⁺ content was assessed in primary patient U-CLL (n = 13) and M-CLL cells (n = 12). **h** Primary patient U-CLL (n = 10-12) and M-CLL cells (n = 10-12) were pretreated for 24 h with ibrutinib (Ibr, 10 µM) and subsequently treated with 500 nM ML162, and specific cell death and lipid peroxidation were assessed. **i** The expression of key pro- and anti-ferroptotic proteins was compared in primary patient U-CLL (n = 11–17) and M-CLL cells (n = 12–17) on the basis of the median fluorescence intensity (MdFI). The data are shown as the fold change between M-CLL/U-CLL cells, and significantly altered proteins are highlighted in red. Statistical significance was determined on the basis of groupwise comparisons of MdFI values. **j** TFRC/CD71 protein levels in primary patient U-CLL and M-CLL cells were analyzed via data retrieved from the proteome dataset by Meier-Abt et al. **k** The left panel shows a representative FACS-based gating strategy for B-cell subsets in HD-derived peripheral blood (n = 10): naïve B cells (NaB), transitional B cells (TrB), non-switched memory B cells (NSM), and switched memory B cells (SM). The TFRC/CD71 levels of the corresponding B-cell subsets measured by FACS are summarized in the right panel. **l** Kaplan‒Meier analysis of treatment-free survival in CLL patients stratified by TFRC/CD71 expression levels, which was performed on the basis of publicly available proteome data from Meier-Abt et al. Statistical analysis: Unpaired t-tests were applied for comparisons between independent groups (Fig. 4b–j), whereas one‒way ANOVA with multiple comparisons was used to assess differences across multiple treatment conditions (**a**, **k**). Kaplan–Meier survival analysis was applied for survival comparisons (**l**). Abbreviations: ‘n’ indicates the sample number; bars represent the mean; P-value: *P < 0.05; **P < 0.01; ***P < 0.001

We first evaluated the sensitivity of these groups to GPX4 inhibition. The results revealed that the IGHV mutational status was a key determinant of sensitivity, with mutated CLL cells being significantly more responsive to ferroptosis induction than unmutated cells (Fig. 4a, Supplementary Fig. 5a, b). Both basal and GPX4 inhibition-induced lipid peroxidation levels were greater in M-CLL cells than in U-CLL cells (Fig. 4b, c). Additionally, Fe^2+^ levels were elevated in M-CLL cells, and the reduction in Fe^2+^ content (e.g., via oxidation to Fe^3+^) following GPX4 inhibition was significantly more pronounced in these cells (Fig. 4d).

In contrast, no significant differences in total intracellular or mitochondrial ROS levels were observed among CLL cell lines with different IGHV mutational statuses, whereas GSH and total NADP(H) levels were lower in the M-CLL lines (Supplementary Fig. 5c–f). When we analyzed primary CLL samples, we confirmed the increased sensitivity of M-CLL cells to ferroptosis and detected similarly higher baseline lipid peroxidation and Fe^2+^ levels in M-CLL cells than in U-CLL cells (Fig. 4e–g, Supplementary Fig. 5g). We also evaluated metabolic differences, such as respiratory activity, as potential contributors to our observations but found no indication of increased mitochondrial activity in M-CLL cells, which could explain their increased susceptibility to ferroptosis via increased mitochondrial ROS release (Supplementary Fig. 5h, i). However, as previously shown,^28^ U-CLL cells display increased basal glycolytic activity and glycolytic reserve, which may contribute to their reduced ferroptosis sensitivity (Supplementary Fig. 5j, k). Elevated glycolytic activity supports antioxidant defense through increased NADPH availability, which is required for the regeneration of reduced GSH. In addition, pyruvate, a key glycolytic intermediate, can directly scavenge ROS and thereby mitigate oxidative stress.^29^ Interestingly, we found that pretreatment with ibrutinib equalized the difference in sensitivity to GPX4 inhibition-induced ferroptosis between U-CLL and M-CLL cells (Fig. 4h).

A subsequent comparative analysis of ferroptosis-related proteins in U-CLL and M-CLL cells revealed increased expression of TFRC/CD71, FTL, and DMT1 in M-CLL cells, all of which play critical roles in iron metabolism (Fig. 4i, Supplementary Fig. 5l). Notably, the increased expression of TFRC/CD71 aligns well with our findings and likely contributes to the increased sensitivity of M-CLL cells to ferroptosis. In support of this, proteomic analyses of CLL cells by Meier-Abt et al.^30^ and Herbst et al.^31^ also confirmed the increased expression of TFRC/CD71 in primary M-CLL cells (Fig. 4j, Supplementary Fig. 5m).

When healthy donor-derived B cells at different stages of differentiation (e.g., naïve, transitional, non-switched memory, and switched memory B cells) were compared, switched memory B cells presented the highest TFRC/CD71 expression. Importantly, switched memory B cells are almost always IGHV-mutated, as they arise from germinal center reactions where somatic hypermutation and affinity maturation occur (Fig. 4k).

Finally, using publicly available datasets from Meier-Abt et al.^30^ and Herbst et al.,^31^ we analyzed the impact of CD71 expression on treatment-free survival (TFS) in CLL patients. Patients with lower CD71 expression, reflecting reduced ferroptosis sensitivity, presented significantly shorter TFS than did those with higher CD71 expression (Fig. 4l, Supplementary Fig. 5n).

### Promoting U-CLL cell sensitivity to ferroptosis by interfering with iron homeostasis and lipid metabolism

**Fig. 5:**
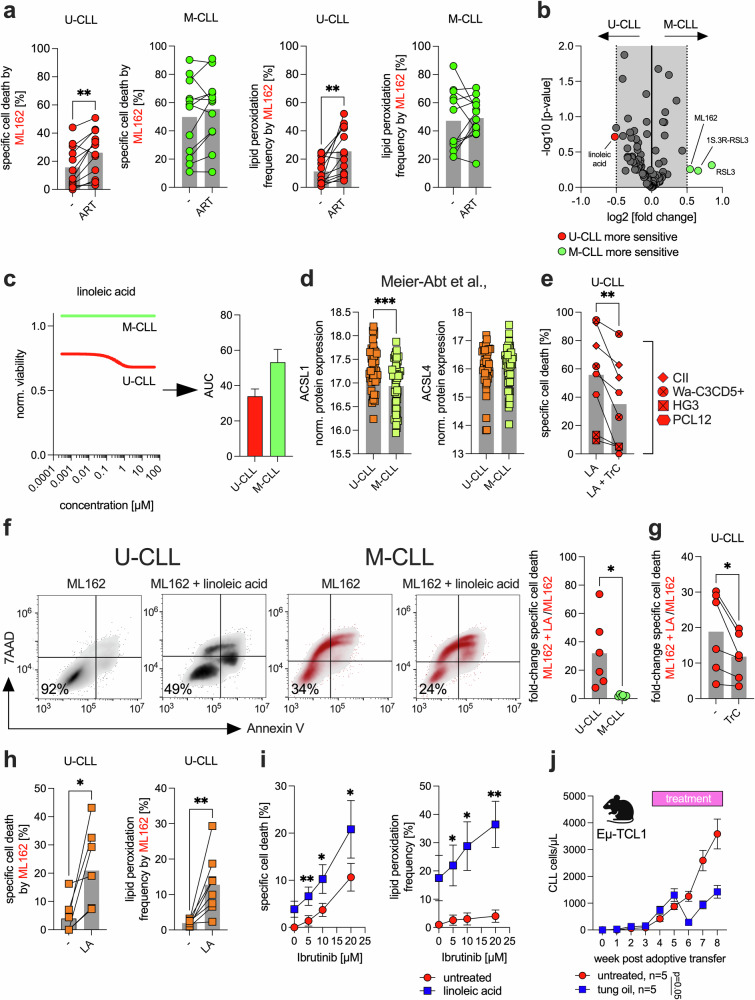
Modifying CLL cell sensitivity toward ferroptosis. **a** U-CLL (CII, HG3, PCL-12, and Wa-C3CD5+) and M-CLL (I83-E95, JVM-3, MEC-1, and PGA-1) cell lines were cultured for 24 h in three independent experiments in the absence (-) or presence (ART) of 10 µM artemisinin and then treated with 100 nM ML162, followed by an assessment of specific cell death and lipid peroxidation. Specific cell death was calculated relative to that of the control (=baseline) as follows: 100 × (% dead cells − % baseline)/(100 − % baseline). The baseline values were normalized to 0%. **b** U-CLL (HG3, CII) and M-CLL (Mec-1, I83-E95) cell lines were utilized in a drug screen with ferroptosis-related compounds at multiple dosages. For each compound, the median viability over all tested concentrations was calculated and expressed as a log₂-fold change comparing U-CLL to M-CLL cell lines, along with the corresponding −log₁₀ p-value. The volcano plot illustrates compounds that promote ferroptosis, with those exhibiting a significantly greater effect on U-CLL cell lines highlighted in red and those with a greater effect on M-CLL cell lines highlighted in green. **c** The dose‒response curve for linoleic acid is shown for the U-CLL (red) and M-CLL (green) cell lines (left panel). The corresponding area under the curve (AUC) is presented in the right panel. **d** ACSL1 and ACSL4 protein levels in primary patient U-CLL and M-CLL cells were analyzed via data retrieved from the proteome dataset by Meier-Abt et al. **e** U-CLL cell lines (HG3, CII, Wa-C3CD5+, and PCL-12) were cultured in two independent experiments for 24 h with or without linoleic acid (LA, 10 µM). Where indicated, Triacsin C (TrC, 5 µM) was added in combination with LA. Specific cell death was assessed by FACS. **f** The left panel shows a representative FACS-based viability analysis of U- and M-CLL cell lines that were cultured for 24 h in the absence or presence of linoleic acid (LA, 10 µM) and then treated with 100 nM ML162. The fold change in specific cell death in linoleic acid (LA)-pretreated cells compared with that in non-pretreated cells is summarized in the right panel for the U-CLL (HG3, CII) and M-CLL (Mec-1, I83-E95) cell lines. Each cell line was assessed in three independent experiments. **g** U-CLL cell lines (HG3, CII) were cultured in three independent experiments for 24 h in the absence or presence of linoleic acid (LA, 10 µM) and then treated with 100 nM ML162. Where indicated, Triacsin C (TrC, 5 µM) was added in combination with LA. The fold change in specific cell death in linoleic acid (LA)-pretreated cells compared with that in non-pretreated cells ± TrC is summarized in the panel. **h** Primary patient U-CLL cells (n = 8) were cultured for 24 h in the absence or presence of linoleic acid (LA, 10 µM) and then treated with 500 nM ML162. Specific cell death and lipid peroxidation were assessed by FACS. **i** U-CLL cell lines (CII, HG3, PCL-12, and Wa-C3CD5+) were treated in two independent experiments with linoleic acid (LA, 10 µM) and increasing dosages of the BTK inhibitor ibrutinib (0–25 µM). Specific cell death and lipid peroxidation were assessed by FACS. **j** Splenocytes from transgenic Eµ-TCL1 mice (≥80% CLL cells) were adoptively transferred into wild-type recipients (n = 5 per group) via i.v. injection. Disease burden was monitored over an 8-week period by measuring CLL cell counts (cells/µL) via FACS. Treatment with tung oil (100 µl/day p.o.) was initiated between weeks 3 and 4 (as indicated). Statistical analysis: Paired t-tests were applied for comparisons involving dependent (matched) samples (**a**, **e**, **g**, and **h**). Unpaired t-tests were applied for comparisons between independent groups (**b**, **d**, and **f**). Two-way ANOVA was applied for analysis of experiments involving multiple factors, such as treatment conditions and/or time points (**i**, **j**). ‘n’ indicates the sample number; bars represent the mean; error bars represent the standard error of the mean; P-value: *P < 0.05; **P < 0.01; ***P < 0.001

Our data suggest that iron metabolism is a key determinant of ferroptosis sensitivity in CLL cells. To further explore this, we evaluated the effects of artemisinin, a known ferroptosis inducer that acts via intracellular Fe accumulation.^32^ Interestingly, for the first time, we observed that a pro-ferroptotic compound had a stronger effect on U-CLL cells than on M-CLL cells (Fig. 5a). This finding may indicate a saturation effect in M-CLL cells, where iron-mediated ferroptosis cannot be further enhanced by increasing Fe^2+^ availability.

Next, we aimed to identify additional pro-ferroptotic molecules that exhibit differential effects on U-CLL (i.e., HG3, CII) and M-CLL (i.e., Mec-1, I83-95) cells. Using a commercially available compound library containing ~100 pro-ferroptotic compounds, we found that linoleic acid had a markedly stronger effect on U-CLL cells (Fig. 5b). Further analysis via area under the curve (AUC) measurements revealed notable differences in overall responsiveness between the U-CLL and M-CLL cell lines. Specifically, M-CLL cells presented numerically higher AUC values than U-CLL cells did when treated with increasing concentrations of linoleic acid (Fig. 5c), suggesting that U-CLL cells respond more strongly to linoleic acid treatment.

The key mediators of linoleic acid-driven ferroptosis include acyl-CoA synthetase long-chain family member 1 (ACSL1) and ACSL4.^33,34^ Interestingly, proteomic data from Meier-Abt et al.^30^ and Herbst et al.^31^ confirmed stronger expression of ACSL1, but not ACSL4, in U-CLL cells (Fig. 5d, Supplementary Fig. 6a). Notably, neither p53 mutation nor 17p deletion influenced ACSL1 expression (Supplementary Fig. 6b, c). Treatment with α-ESA, which is metabolized into linoleic acid, induced specific cell death in U-CLL cell lines. This effect was partially reversed by Triacsin C, a pharmacological inhibitor of both ACSL1 and ACSL4 (Fig. 5e). Consistent with the observation that ACSL1, but not ACSL4, is more strongly expressed in U-CLL cells than in M-CLL cells, α-ESA treatment led to more pronounced sensitization toward ML162-induced cell death, lipid peroxidation, and Fe²⁺ depletion in U-CLL cells than in M-CLL cells (Fig. 5f, Supplementary Fig. 6d, e). The enhanced ML162-induced cytotoxicity in U-CLL cells was likewise reversed by Triacsin C, further supporting the functional role of ACSL1 in mediating ferroptosis sensitivity (Fig. 5g). Furthermore, in primary U-CLL samples, the cytotoxic effects of GPX4 inhibition were significantly enhanced by linoleic acid treatment, and the same effect was observed with ibrutinib (Fig. 5h, i). Finally, in our TCL1 mouse model, we investigated the oral administration of tung oil, which is naturally rich in α-ESA,^33^ and found that it significantly limited disease progression (Fig. 5j).

## Discussion

**Fig. 6:**
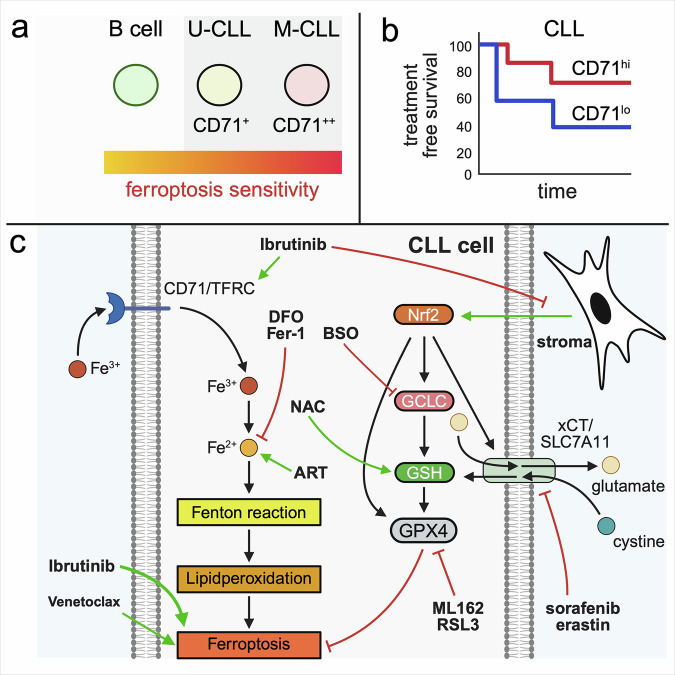
Graphical summary of ferroptosis regulation in CLL. **a** Schematic representation of ferroptosis sensitivity in healthy B cells, unmutated IGHV CLL (U-CLL), and mutated IGHV CLL (M-CLL), highlighting increased CD71 (TFRC) expression and ferroptosis susceptibility. **b** Treatment-free survival of CLL patients stratified by CD71 expression, indicating improved prognosis in CD71^hi(gh)^ patients. **c** Proposed model of ferroptosis regulation in CLL cells. Iron uptake via transferrin receptor 1 (TFRC/CD71) increases intracellular Fe²⁺ levels, fueling the Fenton reaction and lipid peroxidation. GPX4 detoxifies lipid hydroperoxides via glutathione (GSH), whose synthesis depends on GCLC and cystine import via the cystine/glutamate antiporter xCT (SLC7A11), whereas N-acetylcysteine (NAC), a cysteine precursor, can likewise replenish intracellular GSH levels. Stromal contact induces the transcription factor Nrf2, promoting the expression of antioxidant genes, such as GPX4, GCLC, and xCT. Ferroptosis-inducing compounds (e.g., ART, ML162, RSL3, BSO, erastin, and sorafenib) target various nodes of this pathway. Ferroptosis inhibitors (e.g., DFO and Fer-1) block lipid peroxidation or iron redox cycling. Both ibrutinib and venetoclax induce ferroptosis, with ibrutinib exerting stronger effects and additionally acting as a sensitizer by upregulating CD71 and antagonizing stromal protection. CLL chronic lymphocytic leukemia, TFRC transferrin receptor 1, GPX4 glutathione peroxidase 4, GSH glutathione, GCLC glutamate-cysteine ligase catalytic subunit, xCT cystine/glutamate antiporter, SLC7A11 solute carrier family 7 member 11, NAC N-acetylcysteine, Nrf2 nuclear factor erythroid 2–related factor 2, ART artemisinin, BSO buthionine sulfoximine, DFO deferoxamine, Fer-1 ferrostatin-1. The schematic illustration was created with BioRender.com

In our study, we demonstrate that CLL cells are inherently sensitive to the non-apoptotic form of cell death known as ferroptosis and delineate a set of underlying mechanisms that uncover actionable vulnerabilities, as summarized in Fig. 6. Interestingly, ferroptosis can be induced in CLL cells via GPX4 inhibition, similar to what has been reported for DLBCL.^11,12^ However, the inhibition of the xCt cystine/glutamate antiporter failed to trigger ferroptosis, despite its recognized role as a key regulator of this process.^35^ This likely reflects the inherently limited capacity of CLL cells to import cystine, a phenomenon previously described in earlier studies.^15^ Notably, this limited uptake capacity explains the more rapid depletion of intracellular GSH levels observed ex vivo in CLL cells than in healthy B cells,^36^ as cystine is required for GSH synthesis following its conversion to cysteine.

On the other hand, we and others have shown that absolute GSH levels in CLL cells are elevated compared with those in healthy B cells,^6^ which aligns well with the increased expression of the rate-limiting enzyme in GSH synthesis, GCLC. Overall, CLL cells appear to be dependent not only on BCL2 for survival but also on antioxidant defense mechanisms,^37^ with GSH playing a pivotal role. This dependence likely arises from the persistent intrinsic oxidative stress that CLL cells are subjected to, which is partly due to enhanced respiration and concomitant ROS production.^6^ Consequently, targeted GSH depletion can be exploited in vitro to eliminate CLL cells.^38^

In addition to ROS accumulation, CLL cells also exhibit increased lipid uptake,^13,39^ which, in combination with lipid peroxidation, may increase their vulnerability to ferroptosis. Indeed, we observed higher levels of lipid peroxides in CLL cells than in B cells, further supporting their intrinsic sensitivity to ferroptosis. Since iron is essential for lipid peroxidation and thus central to ferroptosis, iron chelators such as DFO exert protective effects. In line with these findings, we detected increased expression of DMT1,^40^ a key Fe^2+^ transporter, along with higher intracellular Fe^2+^ levels in CLL cells than in healthy B cells.

Iron is essential for B-cell function, as deletion of the transferrin receptor TFRC/CD71 in B cells results in substantial functional impairments,^41^ including defects in proliferation and antibody production.^42^ While most circulating CLL cells are quiescent, those residing in lymphoid niches proliferate actively.^43^ Within these protective niches, CLL cells are shielded from intrinsic and extrinsic stressors, including therapy-induced cytotoxicity.^7,8,14^ Accordingly, we observed that stromal interactions enhanced the resilience of CLL cells to ferroptosis. Notably, increased GSH synthesis plays a crucial role in this process, which is mediated by upregulated expression of GCLC and xCt. Additionally, previous studies have shown that stromal cells convert cystine to cysteine, which is subsequently taken up by CLL cells, further enhancing their antioxidant capacity.^15^ Moreover, in addition to mesenchymal stromal cells, other microenvironmental cell types, such as T cells, monocyte-derived nurse-like cells, or endothelial cells, also perform nurturing functions that support CLL survival.^44^ Future studies should therefore explore potential anti-ferroptotic mechanisms involved in these heterocellular interactions.

BTK and BCL2 inhibitors form the backbone of current CLL therapies.^1^ Previous studies in aggressive lymphomas and acute myeloid leukemia (AML) have suggested that both drug classes exert some of their cytotoxic effects through ferroptosis induction.^16,45^ We confirmed this phenomenon in CLL cells and further demonstrated that the first-generation BTK inhibitor ibrutinib enhances ferroptosis sensitivity. Ibrutinib is known to affect cellular energy metabolism^,^^17,18^ and by reducing glycolysis,^46^ it may limit NADPH regeneration and lower intracellular pyruvate levels, both of which are key components of the cellular antioxidant defense. Thus, reduced glycolytic capacity may further sensitize CLL cells to ferroptosis by diminishing their ability to buffer oxidative damage. This finding is further supported by our observation that ibrutinib treatment led to an increase in the intracellular ROS level, a phenomenon previously reported,^47^ and that co-treatment with the antioxidant NAC partially rescued ferroptosis.

Moreover, our findings indicate that ibrutinib also influences iron metabolism. Specifically, ibrutinib treatment led to increased TFRC/CD71 expression and elevated intracellular Fe^2+^ levels.^48^ Importantly, blockade of TFRC/CD71 via specific antibodies significantly attenuated ibrutinib-induced ferroptosis sensitization, highlighting the need for further mechanistic studies to fully elucidate this effect. In this context, we observed the coordinated upregulation of several canonical IRP target genes, such as FTMT, FTH1, and HEPH.^49^ These findings suggest that ibrutinib may induce a functional iron stress response. This could mechanistically result from ibrutinib-induced mitochondrial dysfunction and redox imbalance, both of which are known to activate IRP1/2. Although the exact role of metabolic versus signaling effects is unclear, these findings provide new insights into how BTK inhibition affects iron metabolism in malignant B cells. Future studies will aim to dissect these mechanisms more systematically.

As proof of principle, we evaluated the combination of ibrutinib and the GPX4 inhibitor RSL3 in a preclinical TCL1 mouse model of CLL.^20^ The combination therapy proved to be significantly more effective than monotherapy with either ibrutinib or RSL3, supporting the potential clinical relevance of ferroptosis induction as a therapeutic strategy in CLL.

Genetic features play pivotal roles in CLL prognosis and treatment selection. TP53 mutation status and unmutated IGHV status are well-established negative prognostic markers.^5^ Interestingly, the tumor suppressor p53 is known to promote ferroptosis by downregulating xCt expression.^27^ However, we did not observe a significant impact of TP53 status on ferroptosis sensitivity in CLL. In contrast, IGHV mutational status was a strong determinant, with U-CLL cells being markedly more resistant to ferroptosis than their mutated counterparts (i.e., M-CLL cells). Differences in iron metabolism appear to underlie this discrepancy, as M-CLL cells presented increased expression of TFRC/CD71 and DMT1, leading to increased intracellular Fe^2+^ levels. Nevertheless, considerable heterogeneity in ferroptosis sensitivity was observed even within genetically defined subgroups, suggesting that additional factors, such as redox status, metabolic activity, or the cell cycle phase, may modulate susceptibility and merit further investigation.

Interestingly, among the different B-cell subsets, switched memory B cells presented the highest TFRC/CD71 expression. These cells, which primarily produce IgG, IgA, or IgE rather than IgM, are typically IGHV-mutated and function as key players in adaptive humoral immunity.^50,51^ The increased iron demand in these cells might explain their increased TFRC/CD71 expression, a feature they share with M-CLL cells. Prognostically, higher TFRC/CD71 expression, and consequently increased ferroptosis sensitivity, is correlated with better clinical outcomes, reinforcing the link between iron metabolism and CLL prognosis.^31^ Our findings are in line with recent bioinformatic analyses suggesting that high expression of ferroptosis-promoting genes is associated with a favorable prognosis in CLL.^52^

However, pretreatment with ibrutinib led to an almost equal response of U-CLL and M-CLL cells to ferroptosis induction via GPX4 inhibition. Overall, these observations align well with long-term data from ibrutinib-treated CLL patients, in which ibrutinib-based therapy substantially abrogated the negative prognostic impact of unmutated IGHV status.^53,54^

Given the central role of iron metabolism in determining ferroptosis sensitivity in CLL, we explored pharmacological interventions targeting iron homeostasis. Artemisinin, an antimalarial drug known to increase intracellular labile Fe^2+^ levels by mobilizing stored iron, enhances ferroptosis.^32^ Its derivative, artesunate, has demonstrated activity against B-cell malignancies.^55^ Notably, artemisinin was the first compound we tested that exhibited a stronger pro-ferroptotic effect on U-CLL cells than on M-CLL cells. This observation suggests that in M-CLL, Fe^2+^-mediated ferroptosis may have already reached a steady-state limit.

A subsequent compound screen identified linoleic acid as a potent ferroptosis inducer, particularly in U-CLL cells. Recent studies in breast cancer have shown that linoleic acid induces ferroptosis through ACSL1-mediated incorporation into membrane lipids, promoting lipid peroxidation without directly inhibiting GPX4.^33^ Notably, ACSL1 expression was elevated in U-CLL cells, likely explaining their increased sensitivity to linoleic acid.

To date, no compound has been clinically tested with the specific aim of inducing ferroptosis in CLL. However, several agents already approved for other indications, such as sorafenib, sulfasalazine, gemcitabine, cisplatin, or BSO, have been shown to promote ferroptosis as an additional mechanism of action.^56^ While our data suggest that CLL cells are intrinsically more vulnerable to ferroptosis than healthy B cells are, likely due to disease-associated metabolic reprogramming,^6^ the systemic administration of these agents may pose a risk of off-target toxicity in nonmalignant tissues. To mitigate this, targeted delivery approaches, such as antibody–drug conjugates or nanoparticle-based formulations, may offer safer and more selective means of inducing ferroptosis in leukemic cells.

Importantly, we identified ibrutinib as a pharmacologic ferroptosis sensitizer that reverses stroma-mediated protection, thereby increasing ferroptotic cell death even within typically protective niches such as lymph nodes or bone marrow.^7^ This may support deeper therapeutic responses and facilitate minimal residual disease negativity, a key prognostic goal in CLL.

Furthermore, combining ferroptosis sensitization with T-cell-based immunotherapies, such as chimeric antigen receptor (CAR) T cells,^57^ represents a particularly promising strategy. Activated T cells can induce ferroptosis in tumor cells via interferon-γ secretion.^58,59^ In this context, ibrutinib may act synergistically by lowering the ferroptosis threshold of CLL cells, thereby increasing the efficacy of CAR-T-cell-mediated cytotoxicity. Notably, clinical studies have demonstrated the feasibility and efficacy of combining ibrutinib with CAR-T cells in CLL,^60^ although these investigations did not evaluate ferroptosis as a contributing mechanism. Our findings provide a rationale for further exploration of this axis in future translational studies.

All comparative experiments in this study were performed under identical culture conditions; thus, all CLL and control samples were exposed to the same glucose concentration, which corresponds to hyperglycemic conditions compared with the physiological levels in humans. We therefore consider it unlikely that glucose levels selectively influenced our findings. Moreover, a substantial proportion of our data was obtained directly from primary ex vivo CLL samples or from proteomic and transcriptomic analyses performed without prior cell culture. Nevertheless, we acknowledge that metabolic parameters, including glucose concentration, may modulate ferroptosis-related processes and should be considered in future experimental designs.

In summary, our study highlights ferroptosis as a potential vulnerability in CLL and highlights the critical role of iron metabolism in determining ferroptosis sensitivity. The differential response of U-CLL and M-CLL cells to ferroptosis-inducing agents provides a rationale for further exploration of iron-targeting strategies as a therapeutic approach in CLL.

## Materials and methods

### Primary cells

Blood samples were retrieved from untreated CLL patients upon informed consent in accordance with the Declaration of Helsinki, and patient characteristics are summarized in Supplementary Table 1. Approval numbers of local ethics committees are 289_16B (Friedrich-Alexander University Erlangen-Nuremberg) and 61/22 (Otto-von-Guericke University Magdeburg). Healthy donor (HD) samples were provided by the German Red Cross blood bank. Peripheral blood mononuclear cells (PBMCs) were isolated via Pancoll (PAN Biotech, Germany). B cells were purified via the Human B-Cell Isolation Kit II (Miltenyi Biotec, Germany) following the manufacturer’s manual. Primary B and CLL cells were cultured in AIM-V medium (Thermo Fisher Scientific) under serum-free conditions unless otherwise specified. Primary mesenchymal stromal cells (pMSCs) were isolated from iliac crest bone marrow aspirates of HDs via density gradient centrifugation and expanded in StemMACS MSC Expansion Medium (Miltenyi Biotec), which uniformly fulfilled the minimal criteria.^61^ The approval number of the local ethics committee is 4777 (Friedrich-Alexander University Erlangen-Nuremberg).

### Cell lines

HS-5 and HS-27A cells were obtained from ATCC (VA, USA), and CII, HG3, I83-E95, JVM-3, Mec-1, PCL-12, PGA-1, and Wa-C3CD5+ cells were obtained from DSMZ GmbH (Germany). The cells were maintained in culture with RPMI-1640 (Thermo Fisher Scientific, MA, USA) supplemented with 10% FBS (FBS High Performance, Bio&Sell, Germany), 2 mM L-glutamine (Thermo Fisher Scientific), and 40 U/mL Pen/Strep (Merck, Germany). The authenticity of the cell lines was regularly and most recently verified in January 2025 via STR profiling (Eurofins Scientific SE, Luxembourg, or Microsynth AG, Switzerland). Cultures were routinely tested for mycoplasma contamination via in-house PCR analysis (Minerva Biolabs, Germany).

### Ferroptosis induction

The cells were cultured in serum-free AIM-V medium (Thermo Fisher Scientific) supplemented with 3 g/l glucose at 37 °C and 5% CO₂. For the selected experiments, ferroptosis induction was performed for 24–48 h in cocultures with the human bone marrow stroma cell lines HS-5 or HS-27A and with healthy donor-derived pMSCs. A comprehensive list of compounds and chemicals is provided in Supplementary Table 2.

### Multiparametric flow cytometry

For flow cytometry (FACS) analyses, samples were stained with fluorochrome-conjugated antibodies and dyes as described below or in the Supplementary Information, and a complete list can be found in the Supplementary Tables 3 and 4. The samples were recorded on a Cytek NL-3000 full-spectrum flow cytometer (Cytek Biosciences, CA, USA). The data were analyzed via FlowJo Version 10 (FlowJo LLC, OR, USA).

For all the analyses, the cells were stained with a fixable viability dye and treated with human IgG to block nonspecific binding (Gamunex, Grifols, Spain). Next, the cells were incubated either directly or after fixation and permeabilization via a BD Cytofix/Cytoperm™ Fixation/Permeabilization Kit (BD Biosciences, NJ, USA) with antibodies and/or dyes.

### Cell viability

Viability was assessed via FACS by co-staining cells with Annexin V and 7-AAD in Annexin V staining buffer (BioLegend, CA, USA). Specific cell death was calculated as % = 100 × (% death − % baseline death)/(100 − % baseline death).^62^

### Lipid peroxidation

Lipid peroxidation was assessed via FACS using the lipid peroxidation sensor BODIPY™ 581/591 C11 (Thermo Fisher Scientific) at a 1:100 dilution. The samples were incubated for 20 min at 37 °C in 5% CO₂. This sensor incorporates polyunsaturated fatty acid analogs, which undergo an emission shift from 590 nm (non-oxidized) to 510 nm (oxidized) upon oxidation. To differentiate between the oxidized and non-oxidized forms, we utilized positive controls.

### Ferrous (Fe^2+^) iron pool

The ferrous iron pool was analyzed via FACS using the fluorescent probe Phen Green SK diacetate (Thermo Fisher Scientific), whose fluorescence signal is quenched by Fe²⁺ ions. As a result, its fluorescence intensity is inversely proportional to the intracellular ferrous iron level. To provide a more intuitive visualization, Fe²⁺ changes upon treatment are expressed as the −log₂ fold change between the untreated and treated conditions. In selected experiments, changes in the intracellular Fe²⁺ pool were independently validated via the use of a BioTracker™ 575 Red Fe²⁺ Dye (Merck), a highly specific fluorescent sensor that increases in signal intensity upon binding to Fe²⁺. Measurements were performed via FACS.

### Reactive oxygen species, cellular antioxidants, and cystine uptake

The levels of mitochondrial superoxides and total cellular reactive oxygen species (ROS) were determined via FACS using MitoSOX^TM^ Red and CellROX^TM^ Deep Red (Thermo Fisher Scientific). Glutathione, which represents the majority of the intracellular thiols, was semiquantified via FACS using ThiolTracker^TM^ Violet (Thermo Fisher Scientific). Cystine uptake was analyzed by FACS using BioTracker Cystine-FITC Live Cell Dye (Merck). The total cellular antioxidative capacity was determined via an antioxidant assay kit (Cayman Chemicals). The total intracellular NADP(H) concentration was quantified via a colorimetric NADP/NADPH assay kit (Abcam).

### CRISPR-Cas9–mediated gene knockout

Target genes were knocked out via the Alt-R™ CRISPR-Cas9 system (Integrated DNA Technologies, Coralville, IA, USA) in combination with the Neon Nxt™ electroporation system (Thermo Fisher Scientific), following the manufacturer’s protocols. In brief, predesigned Alt-R™ CRISPR-Cas9 single guide RNAs (sgRNAs) were complexed with Alt-R™ S.p. Cas9 nuclease V3 to form ribonucleoprotein (RNP) complexes. These were delivered into 1 × 10⁶ cells in a 10 µL total volume by electroporation (one pulse, 1450 V, 20 ms) in the presence of the Alt-R™ Cas9 Electroporation Enhancer. Following electroporation, the cells were cultured in complete RPMI-1640 medium and incubated for 48 h before downstream analysis. The detailed sgRNA sequences are provided in Supplementary Table 5.

### Statistics

Paired or unpaired two-tailed *t*-tests were used as appropriate for comparisons between two groups, depending on whether the measurements were dependent (matched) or independent. For comparisons involving more than two groups, one-way ANOVA or repeated-measures ANOVA was applied, followed by appropriate post hoc testing. Two-way ANOVA was used for experiments involving multiple independent variables, such as treatment conditions and time points. Survival analyses were conducted via Kaplan–Meier curves and compared via the log-rank (Mantel–Cox) test. The outliers were identified via the ROUT method. A p-value < 0.05 was considered statistically significant. The specific statistical tests used for each dataset are indicated in the respective figure legends. All analyses were performed via GraphPad Prism version 10 (GraphPad Software Inc., San Diego, CA, USA).

For more information, see the Supplementary Information.

## Supplementary information

Supplemental Material

## Data Availability

This publication includes additional analyses of previously generated mass spectrometry–based proteomics datasets.^30,31^ The proteomics data were deposited to the ProteomeXchange Consortium via the PRIDE partner repository under the following dataset identifiers: PXD022198 and PXD022216, as well as PXD028936 and PXD024544. No new raw proteomics or single-cell sequencing data were generated for this study. Any additional information is available from the corresponding authors upon reasonable request.
