# Supplementary material for: Immunoglobulin heavy-chain status and stromal interactions shape ferroptosis sensitivity in chronic lymphocytic leukemia
Source: Signal Transduct Target Ther. 2026 Jan 5;11:3. doi: 10.1038/s41392-025-02535-x (PMC12765864; doi:10.1038/s41392-025-02535-x)
Supplement: Supplementary file 1 — Supplemental Material [file 41392_2025_2535_MOESM1_ESM.pdf]

Supplementary Materials for

IGHV status and stromal interaction shape ferroptosis sensitivity in chronic lymphocytic leukemia.

Martin Böttcher*, Lea Reemts, Paul J. Hengeveld, Romy Böttcher-Loschinski, Vikas Bhuria, Junyan Lu, Silvia Materna-Reichelt, Durdam Das, Natasa Stojanović Gužvić, Heiko Bruns, Wolfgang Huber, Thorsten Zenz, Denny Schanze, Martin Zenker, Sascha Dietrich, Anton W. Langerak, Dimitrios Mougiakakos*

*Correspondence to: martin.boettcher@med.ovgu.de & dimitrios.mougiakakos@med.ovgu.de

This PDF file includes:

Materials and Methods
Supplementary Figures S1 to S6
Supplementary Tables S1 to S5

Materials and Methods

TP53 mutational status

To determine the TP53 mutational status, we isolated genomic DNA following standard protocols. Specific oligonucleotide primers were designed using Primer3 software (Primer3web version 4.1.0; https://primer3.ut.ee/) to amplify the 10 coding exons and flanking intronic regions of the TP53 gene. Primer sequences and PCR conditions are available upon request. Mutational analyses were conducted using BigDye Terminator v3.1 chemistry on an ABI 3500xl Dx DNA analyzer (Applied Biosystems, CA, USA). All obtained sequences were compared to the TP53 reference sequence (ENST00000269305.8) using SEQUENCE Pilot software (version 5.4.0; JSI Medical Systems GmbH, Germany). Mutations were designated according to the Human Genome Variation Society (HGVS) guidelines (http://www.hgvs.org/mutnomen/).

Quantitative real-time PCR

Total RNA was isolated using the innuPREP RNA Mini Kit 2.0 (iST Innuscreen GmbH, Germany). Complementary DNA (cDNA) was synthesized using the High-Capacity cDNA Reverse Transcription Kit (Thermo Fisher Scientific), and quantitative PCR (qPCR) was performed with the Luna® Universal qPCR Master Mix (New England BioLabs Inc., MA, USA). All steps were carried out according to the manufacturers' protocols using a primer array for ferroptosis (Human Ferroptosis PCR Primer Library, Real Time Primers LLC, PA, USA). Data acquisition was performed on a QuantStudio 3 qPCR system, and relative gene expression was calculated using the ΔCt method, with NormFinder[1] implemented in Microsoft Excel Version 16 (Microsoft, Redmond, Washington, USA) to identify optimal normalization genes.

Bioenergetic profiling

Real time analyses were performed on the Seahorse XFe 96 (Agilent, CA, USA), as previously described in detail[2]. Data was analyzed using Seahorse Wave Software 2.6.3 and parameters were calculated with Microsoft Excel Version 16 (Microsoft).

Compound screen

A library of 320 compounds from TargetMol (Boston, MA, USA) was provided in 4 × 96-tube racks, each containing 100 µl of 10 mM compound in DMSO. The library was reformatted into a 384-well storage block using an Integra VIAFLO 96/384 pipetting device, then transferred into small-volume storage plates (Greiner Bio-One, Germany) and stored at -80°C. Working stock plates (1, 0.1, 0.05, and 0.00408 mM) were also prepared and stored at -80°C.

For assay preparation, 8 µl of 10 mM, 0.05 mM, and 0.00408 mM stocks were transferred to Echo® 384-well microplates (Greiner Bio-One) and diluted to final concentrations (50 to 0.0003 µM) with 0.52% DMSO. Plates were thermosealed and stored at -20°C.

For drug screening, HG-3, Mec-1, CII, and I-85-E95 cell lines were cultured in RPMI-1640 with 10% FBS, 1% GlutaMAX, and 1% Penicillin/Streptomycin. Before screening, cells were pre-incubated in AIMV medium for 72 h before seeding.

The cell lines were screened at 10 concentrations of the ferroptosis library. To control for plate effects, 2–4 DMSO plates and one tool compound control plate were included per cell line.

On the experiment day, cells were transferred into fresh AIMV medium and seeded (10,000 cells/well in 40 µl) into 384-well assay-ready plates (Greiner Bio-One, Germany) using a Multidrop Combi dispenser. After 24 h incubation at 37°C, 5% CO_2, 20 µl/well of ATPlite 1-Step

Luminescence Assay (Revvity, MA, USA) was added via Multidrop Combi, and viability was measured using an Envision Plate Reader (Revvity).
A total of 320 compounds were screened across four cell lines, along with a positive control (Bortezomib 50 μM) and two tool compounds (RSL3, ML162 at 25–800 nM). DMSO (0.52%) served as a negative control.
Viability (ATPlite readout) and Echo log files were mapped to retrieve plate data and exclude skipped wells, marked as "skipped." Each well contained a single compound at one of 10 tested concentrations. Viability values were normalized using the median DMSO viability for each plate ($norm\_val_x$), calculated as:

$$norm\_val_x = median(DMSO\_plate_x)$$

Post-normalization, viability distributions were analyzed using heatmaps. Spatial effects were observed in all plates and corrected based on DMSO-only plate patterns.
Response to the compounds was evaluated by calculating the median viability of each cell line across tested concentrations using normalized viability values. The $\log_2$ fold change in viability between M-CLL and U-CLL was computed. Statistical significance was assessed using Student's t-test, with p-values represented as $-\log_{10}$(p-value) in the plot.

Animal experiments

All animal experiments were conducted in compliance with the European Union Directive 2010/63/EU and German animal welfare regulations. Experimental protocols were reviewed and approved by the animal ethics committee of the Federal State of Saxony-Anhalt (approval number 2-1746), ensuring adherence to the highest ethical standards. Animals were housed and cared for under conditions that aligned with national and international guidelines for laboratory animal welfare, with every effort made to minimize discomfort and distress throughout the study.
We used the transgenic Eμ-Tcl1 CLL mouse model (C57Bl6/J background) as well as the adoptive transfer model injecting 10^7 splenocytes i.v. per animal into 6-8 weeks old female recipient C57Bl/6J wild type mice[3,4]. Disease progression was monitored weekly in peripheral blood by FACS, assessing $CD19^+/CD5^+$ CLL cell count per μL and their frequency within the $CD45^+$ population. Animals were sacrificed upon reaching the humane endpoint >80% CLL cells in the peripheral blood as required by the local regulatory agency for animal welfare.
Animals in the adoptive transfer model were randomly assigned to different treatment groups (n=5 per group) and received treatment starting between week 3 and 4. The treatment regimens included ibrutinib (0.16 mg/mL in sterile drinking water containing 1% hydroxypropyl-beta-cyclodextrin), RSL3 (10 mg/kg in 100 μL 3% DMSO/97% corn oil, administered twice a week via intraperitoneal injection), a combination of ibrutinib and RSL3, or tung oil (100 μl/day by oral gavage). Vehicle-treated animals served as untreated controls. Treatment was continued until animals were sacrificed.

Supplementary References

1 Andersen, C. et al. Normalization of real-time quantitative reverse transcription-PCR data: a model-based variance estimation approach to identify genes suited for normalization, applied to bladder and colon cancer data sets. Cancer Res 64, 5245-5250 (2004).

2 Lu, J. et al. Energy metabolism is co-determined by genetic variants in chronic lymphocytic leukemia and influences drug sensitivity. Haematologica 104, 1830-1840 (2019).

3 Fernandez Botana, I. et al. Emu-TCL1 adoptive transfer mouse model of chronic lymphocytic leukemia. Methods Cell Biol 188, 109-129 (2024).

4 Bichi, R. et al. Human chronic lymphocytic leukemia modeled in mouse by targeted TCL1 expression. Proc Natl Acad Sci U S A 99, 6955-6960 (2002).

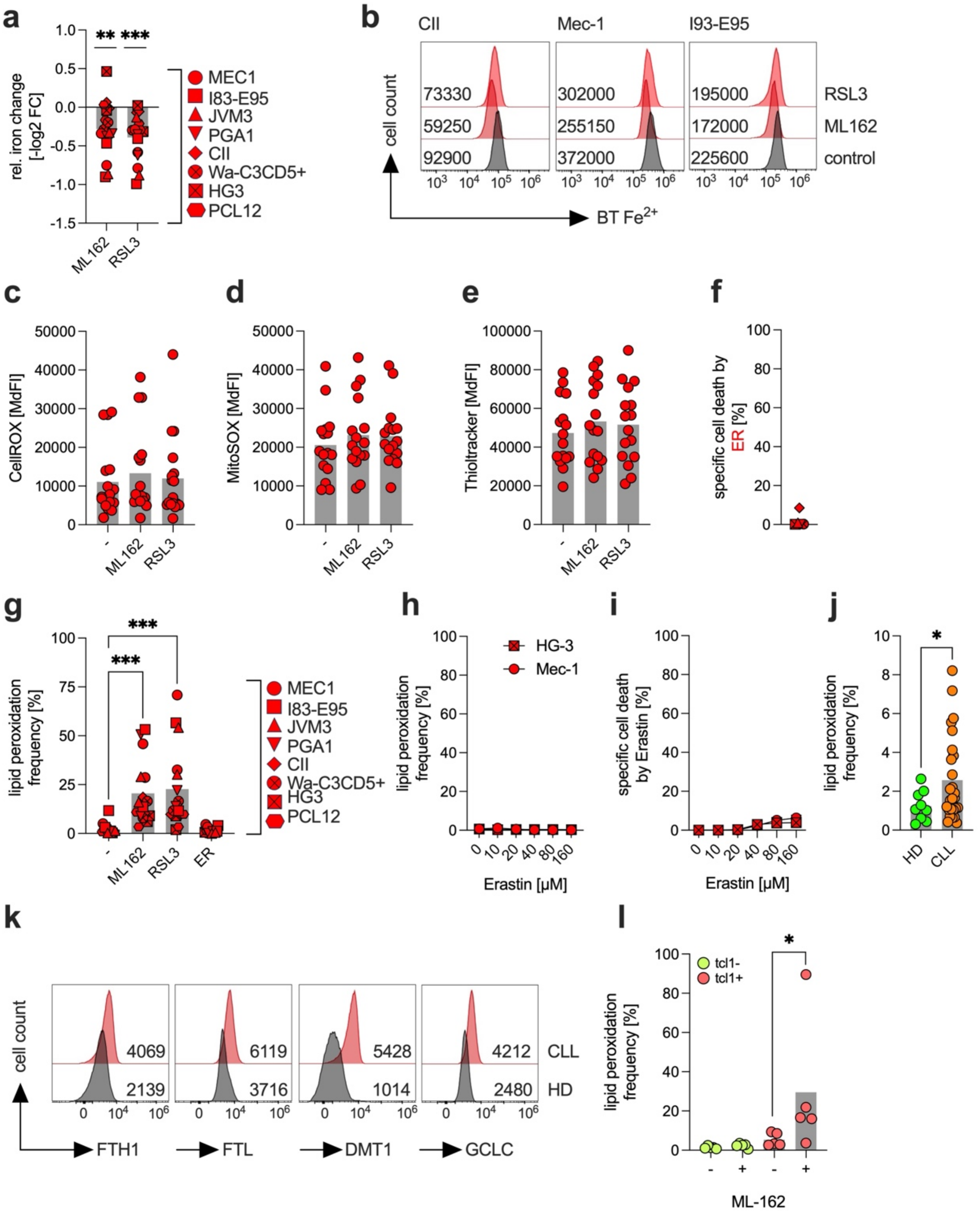

Supplementary Figure 1. The CLL cell lines CII, HG3, I83-E95, JVM-3, Mec-1, PCL-12, PGA-1, and Wa-C3CD5^{+} were treated in three independent experiments with 100 nM of the GPX4 inhibitors ML-162 or RSL3 for 4 h. **(a)** The relative change in intracellular Fe^{2+} levels was assessed by FACS using the fluorescent probe Phen Green SK. **(b)** To validate these findings, intracellular Fe^{2+} levels were also measured using the BioTracker™ Far Red Fe^{2+} dye (BT Fe^{2+}). Representative FACS histograms for three selected cell lines are shown, with values indicating semi-quantified median fluorescence intensity (MdFI). Furthermore, we assessed **(c)** total cellular ROS using CellROX, **(d)** mitochondrial superoxide levels using MitoSOX, and **(e)** intracellular glutathione

content using ThiolTracker, all by FACS. All cell lines were treated in three independent experiments with the xCt inhibitor Erastin (ER, 10 μM) for 4 h and compound-triggered specific cell death **(f)** as well as lipid peroxidation **(g)** were assessed by FACS. Specific cell death was calculated relative to the control (=baseline) using: 100 × (% dead cells − % baseline)/(100 − % baseline). Baseline values were normalized to 0%. Additionally, HG-3 and Mec-1 CLL cells were treated in two independent experiments with increasing concentrations of ER (0-160 μM) for 24 h and compound-triggered specific cell death **(h)** as well as lipid peroxidation **(i)** were assessed. **(j)** Baseline lipid peroxidation of B cells from healthy donors (HD, n=9) and CLL cells from patients (CLL, n=26) was assessed by FACS. **(k)** Representative FACS histograms of significantly differentially expressed proteins (i.e., FTH1, FTL, DMT1, and GCLC) in $CD19^+$ B cells from healthy donors (HD, black) and patient-derived CLL cells (CLL, red), analyzed *ex vivo*. MdFI values are indicated. **(l)** Lipid peroxidation was assessed in CLL/B cells from non-transgenic littermates (TCL1-, n=5) and transgenic Eμ-TCL1 mice (TCL1+, n=5) following *ex vivo* culture for 24 h in absence (-) or presence (+) of 500 nM ML-162. Statistical analysis: Paired t-tests were applied for comparisons involving dependent (matched) samples (Fig. S1a, f, and l). Unpaired t-tests were used for comparisons between independent groups (Fig. S1j). One-way ANOVA with multiple comparisons was used to assess differences across multiple treatment conditions (Fig. S1c-e, S1g). Abbreviations: ‘n’ indicates the sample number; bars represent mean; P value: *P < 0.05; **P < 0.01; ***P < 0.001.

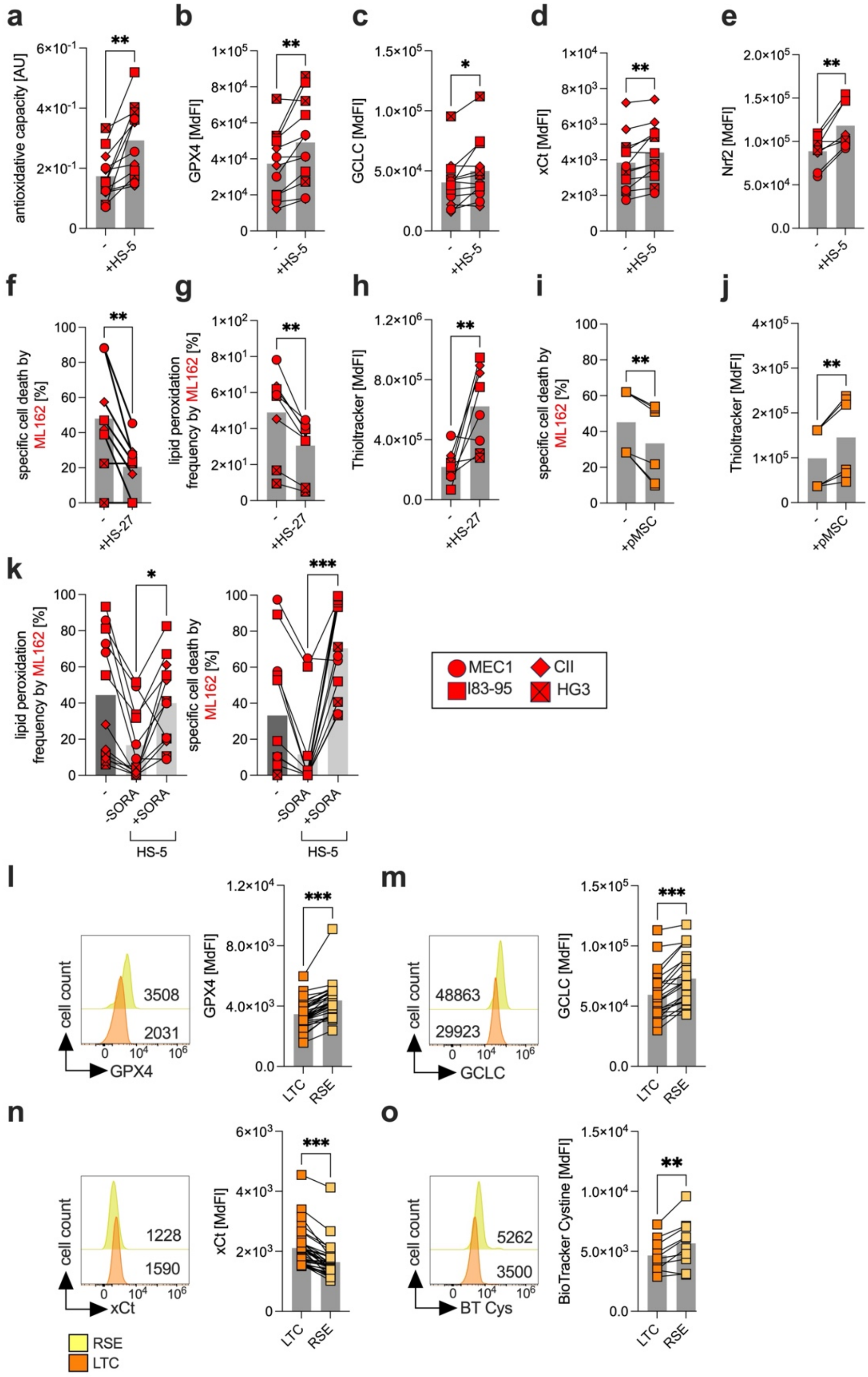

Supplementary Figure 2. The CLL cell lines CII, HG3, I83-E95, and Mec-1 were cultured in three independent experiments for 24 h with or without HS-5. **(a)** Total cellular antioxidative capacity was assessed using an enzymatic microplate-based assay, while protein expression of **(b)** GPX4, **(c)** GCLC, **(d)** xCT, and **(e)** Nrf2 was measured by flow cytometry (FACS). The CLL cell lines CII, HG3, I83-E95, Mec-1were cultured in presence/absence of HS-27 cells for 24 h,

followed by treatment with ML-162 (100 nM) and **(f)** specific cell death, **(g)** lipid peroxidation, and **(h)** intracellular thiols were assessed by FACS. Specific cell death was calculated relative to the untreated control using: 100 × (% dead cells − % baseline)/(100 − % baseline). Baseline values were normalized to 0%. In a separate experiment, primary CLL cells from two patients were co-cultured with or without primary mesenchymal stromal cells (pMSCs) from three healthy donors for 48 h. Cells were then treated with ML-162 (100 nM) for 24 h or left untreated. **(i)** Specific cell death was assessed by FACS. **(j)** Intracellular thiol content was quantified using ThiolTracker via FACS. **(k)** CII, HG3, I83-E95, and Mec-1 cells were co-cultured in three independent experiments with/without HS-5 cells in presence/absence of the inhibitor of cysteine uptake Sorafenib (SORA, 30 μM). Following treatment with 100 nM ML162 lipid peroxidation and specific cell death were assessed by FACS. **(l-o)** Primary CLL cells (n=10-34) were stratified into $CD5^{high}CXCR4^{low}$ recent stromal emigrant (RSE) and $CD5^{low}CXCR4^{high}$ long-term circulating cells (LTC) and analyzed by FACS for **(l)** GPX4, **(m)** GCLC, **(n)** xCt, and **(o)** cystine (i.e., BioTracker Cystine/BT Cys) uptake. Representative FACS histograms are shown for each marker, with values indicating semi-quantified median fluorescence intensity (MdFI). Quantitative data are summarized in the accompanying dot plots. Statistical analysis: Paired t-tests were applied for comparisons involving dependent (matched) samples (Fig. S2a-o). Abbreviations: ‘n’ indicates the sample number; bars represent the mean; P value: *P < 0.05; **P < 0.01; ***P < 0.001.

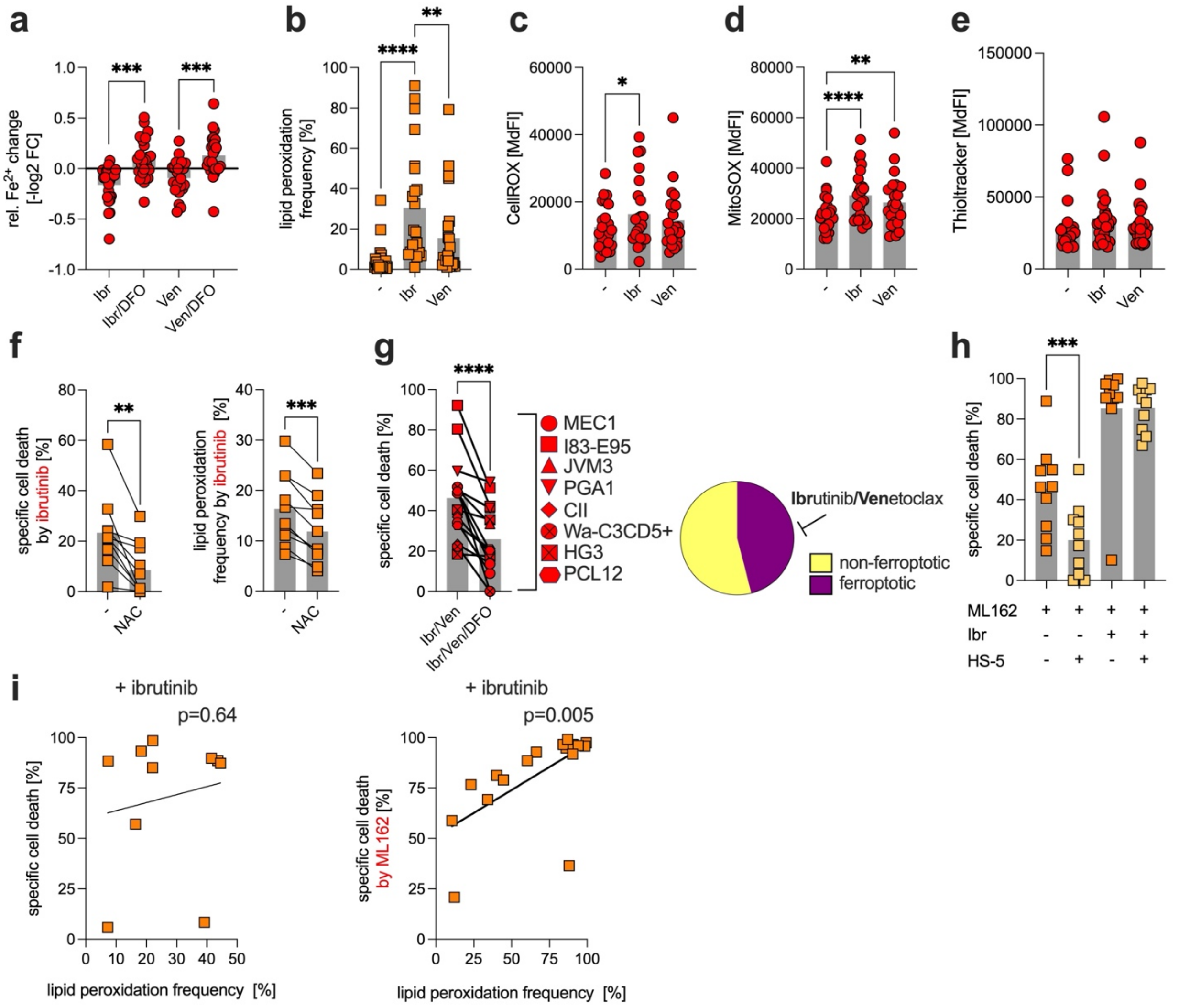

Supplementary Figure 3. (a) The CLL cell lines CII, HG3, I83-E95, JVM-3, Mec-1, PCL-12, PGA-1, and Wa-C3CD5+ were cultured in three independent experiments for 24 h in presence or absence of ibrutinib (Ibr, 10 µM) or venetoclax (Ven, 50 nM), with or without deferoxamine (DFO, 100 µM) and relative change of Fe^{2+} using Phen Green SK was assessed by flow cytometry (FACS). **(b)** Primary patient CLL samples (n=23) were cultured for 24 h in presence or absence of ibrutinib (Ibr, 10 µM) or venetoclax (Ven, 1 nM) and lipid peroxidation was assessed by FACS. **(c-e)** The CLL cell lines CII, HG3, I83-E95, JVM-3, Mec-1, PCL-12, PGA-1, and Wa-C3CD5+ were cultured for 24 h in presence or absence of ibrutinib (Ibr, 10 µM) or venetoclax (Ven, 50 nM) and **(c)** cellular ROS using CellROX, **(d)** mitochondrial superoxides using MitoSOX, and **(e)** glutathione content using ThiolTracker were assessed by FACS. **(f)** Patient-derived CLL cells (n=10, orange squares) were treated for 24 h with ibrutinib (10 µM) in presence or absence of N-acetylcysteine (NAC, 5 µM). Cell viability and lipid peroxidation were assessed by FACS, and specific cell death was calculated accordingly. Specific cell death was calculated relative to the control (=baseline) using: 100 × (% dead cells − % baseline)/(100 − % baseline). Baseline values were normalized to 0%. **(g)** The CLL cell lines CII, HG3, I83-E95, JVM-3, Mec-1, PCL-12, PGA-1, and Wa-C3CD5+ were cultured for 24 h in presence or absence of a combination of ibrutinib (Ibr, 10 µM) and venetoclax (Ven, 50 nM), with or without deferoxamine (DFO, 100 µM). Cell

viability was assessed by FACS, and specific cell death was calculated accordingly (left panel). The contribution of ferroptosis to the overall cytotoxicity induced by ibrutinib/venetoclax is shown (right panel). The calculation is based on the rescue potential of DFO. **(h)** Primary CLL cells (orange squares, n=10) were cultured in presence/absence of HS-5 cells for 48 h, followed by treatment with ML-162 (100 nM) with or without ibrutinib (Ibr, 10 μM). Specific cell death was assessed by FACS. **(i)** Primary CLL cells (n =10, orange squares) were treated with/without ibrutinib (10 μM) for 24 hours (left panel) followed by treatment with 500 nM ML162 (right panel). Specific cell death and lipid peroxidation were assessed by FACS and correlated using linear regression. Statistical analysis: Paired t-tests were applied for comparisons involving dependent (matched) samples (Fig. S3a, f-i). One-way ANOVA with multiple comparisons was used to assess differences across multiple treatment conditions (Fig. S3b-e). Abbreviations: 'n' indicates the sample number; bars represent the mean; P value: *P < 0.05; **P < 0.01; ***P < 0.001.

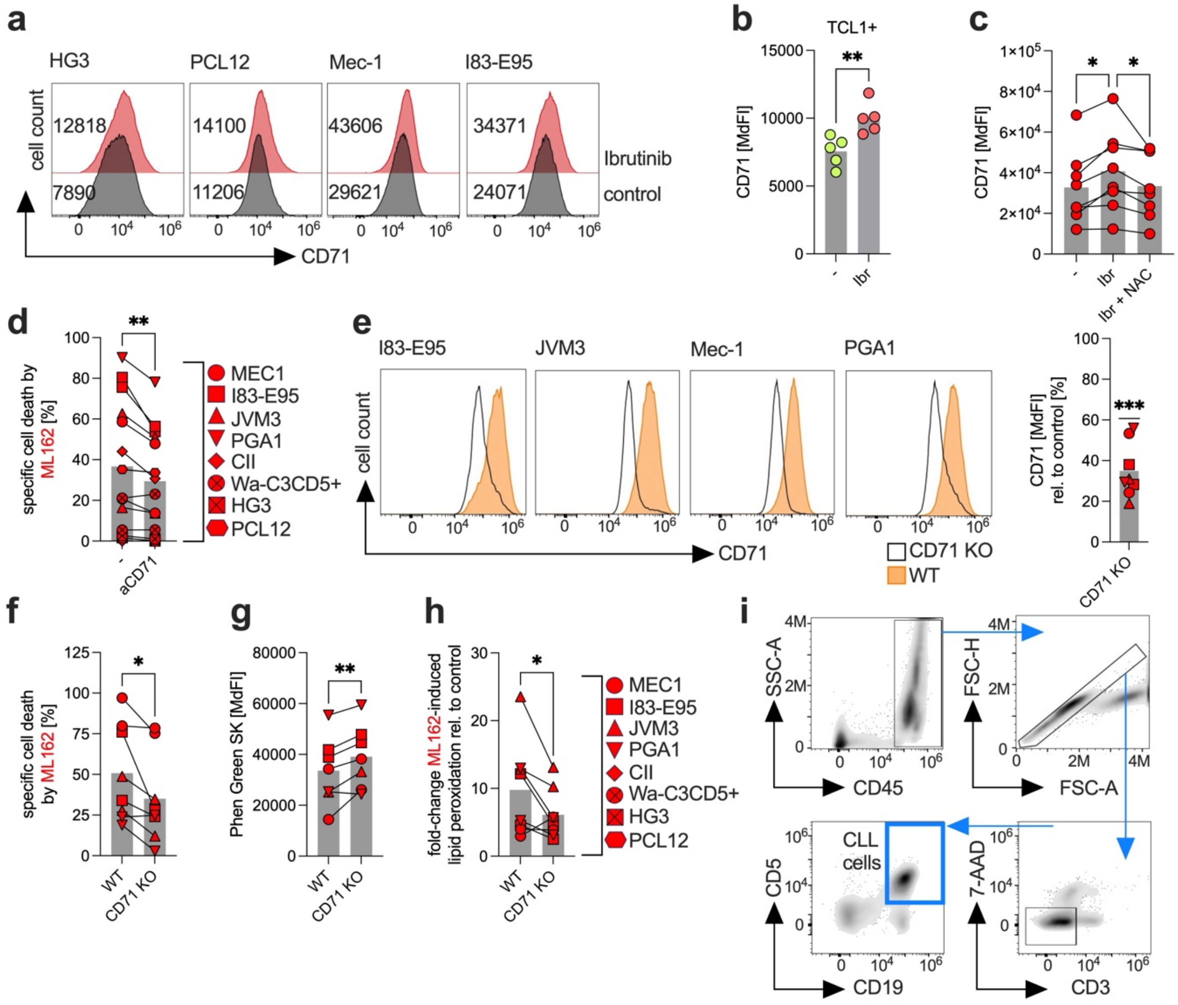

Supplementary Figure 4. (a) Representative FACS histograms of TFRC/CD71 surface expression in four selected CLL cell lines treated with or without ibrutinib. Median fluorescence intensity (MdFI) values are indicated. **(b)** *Ex vivo* analysis of TFRC/CD71 MdFI in CLL cells from C57BL/6 mice after adoptive transfer of splenocytes from Eμ-TCL1 transgenic animals (TCL1+) treated with/without ibrutinib. **(c)** The CLL cell lines CII, HG3, I83-E95, and Mec-1 were cultured in two independent experiments for 24 h in presence or absence of ibrutinib (Ibr, 10 μM) with or without NAC (5 μM) and transferrin receptor (TFRC/CD71) surface protein was measured by FACS. **(d)** The CLL cell lines CII, HG3, I83-E95, JVM-3, Mec-1, PCL-12, PGA-1, and Wa-C3CD5+ were treated in two independent experiments for 24 hours with either an IgG isotype control (-) or an anti-CD71 blocking antibody (aCD71, 2 μg/mL), followed by treatment with 100 nM ML162 and assessment of specific cell death. **(e)** The cell lines I83-E95, JVM3, MEC1, and PGA1 were subjected to CRISPR-Cas9-mediated knockout (KO) of TFRC/CD71. Representative histograms (left panel) show CD71 surface expression in wild-type (WT, orange) and CD71 knockout (CD71 KO, black) cells. The analysis was performed at the bulk population level and all experiments were conducted in biological duplicates. The right panel summarizes CD71 expression based on the MdFI in CD71 KO cells relative to WT controls across the four lines. WT and KO cell lines were treated in two independent experiments with ML-162 (100 nM) for 4 h and **(f)** specific cell death, **(g)** labile iron levels using Phen Green SK, and **(h)** lipid peroxidation determined by FACS. Note that Phen Green SK signal is quenched by Fe^{2+}; thus, higher

fluorescence indicates lower intracellular ferrous iron levels. **(i)** Disease progression in mice was monitored longitudinally by FACS of peripheral blood samples. Representative plots illustrate the gating strategy for CD45 vs. side scatter (SSC), singlets, viable $CD3^{-}$ cells, and malignant $CD19^{+}$/$CD5^{+}$ CLL cells. Statistical analysis: Paired t-tests were applied for comparisons involving dependent (matched) samples (Fig. S4b, and d-h). One-way ANOVA with multiple comparisons was used to assess differences across multiple treatment conditions (Fig. S4c). Abbreviations: 'n' indicates the sample number; bars represent the mean; P value: *P < 0.05; **P < 0.01; ***P < 0.001.

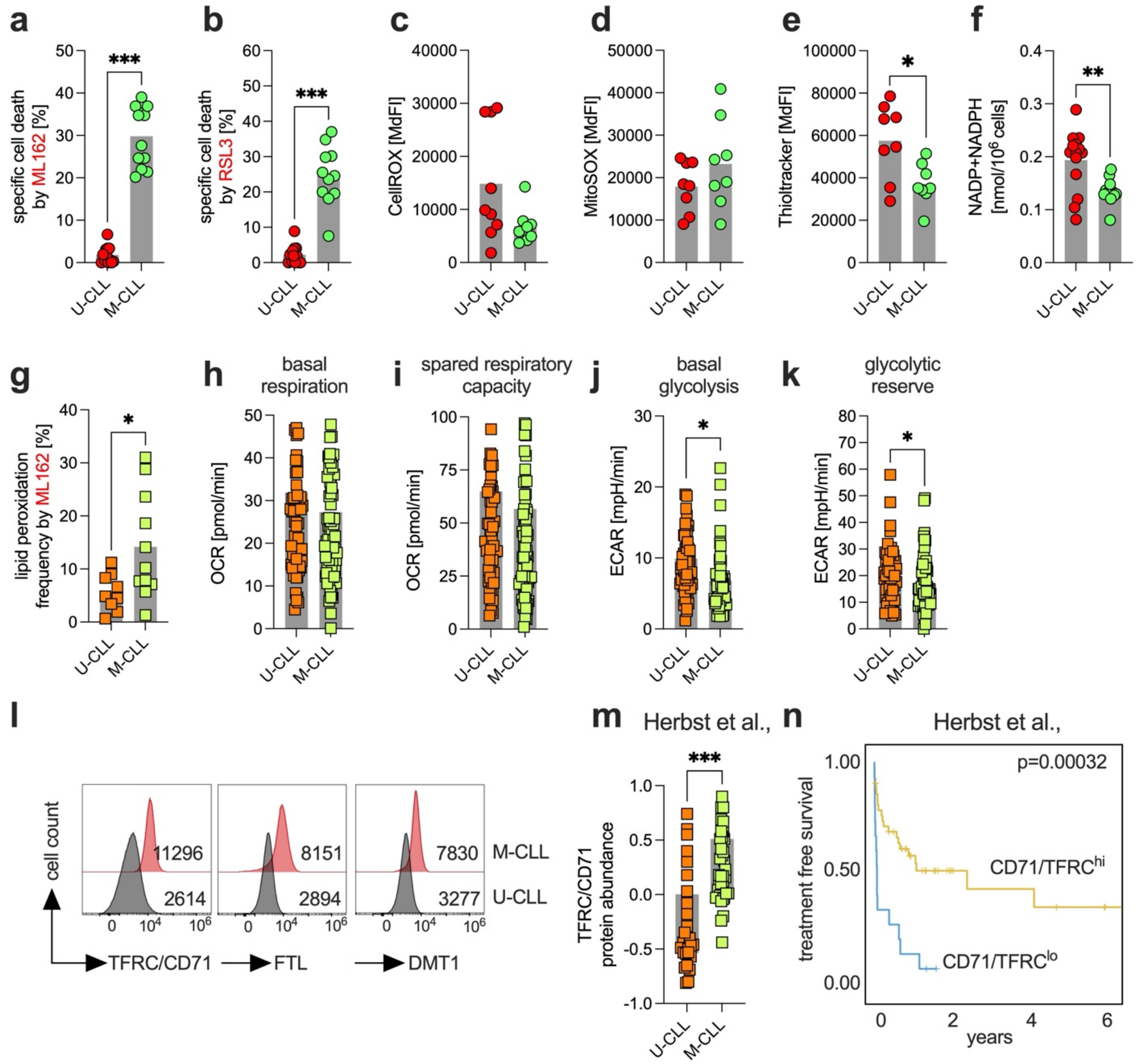

Supplementary Figure 5. U-CLL (CII, HG3, PCL12, and WaC3CD5+) and M-CLL (I83-E95, JVM-3, MEC-1, and PGA1) cell lines were treated with the GPX4 inhibitor **(a)** ML-162 (100 nM) or **(b)** RSL3 (100 nM) for 4 h and compound-triggered specific cell death was determined by flow cytometry (FACS). Specific cell death was calculated relative to the control (=baseline) using: 100 × (% dead cells − % baseline)/(100 − % baseline). Baseline values were normalized to 0%. U-CLL (CII, HG3, PCL12, and WaC3CD5+) and M-CLL (I83-E95, JVM-3, MEC-1, and PGA1) cell lines were analyzed for (**c**) cellular ROS using CellROX, (**d**) mitochondrial superoxides using MitoSOX, (**e**) glutathione content using ThiolTracker by FACS, and (**f**) total NADP(H) content using a fluorometric assay. (**g**) Primary patient U-CLL (n=9) and M-CLL (n=11) cells were treated with 500 nM ML162 and lipid peroxidation was assessed by FACS. Bioenergetic parameters of primary U-CLL (n=51-62) and M-CLL (n=65-77) were determined using bioenergetic analyses (Seahorse, Agilent) and (**h**) basal respiration, (**i**) spare respiratory capacity (SRC), (**j**) basal glycolysis, and (**k**) glycolytic reserve were calculated based on the measured oxygen consumption rate (OCR) and extracellular acidification rate (ECAR), respectively. **(l)** Representative FACS

histograms of significantly differentially expressed proteins (TFRC, FTL, and DMT1) in U-CLL (black) and M-CLL (red) CLL cells. Median fluorescence intensity (MdFI) values are indicated. (**m**) TFRC/CD71 protein levels in primary patient U-CLL and M-CLL cells were analyzed using data retrieved from the proteome dataset by Herbst *et al*. (**n**) Kaplan-Meier analysis of treatment-free survival in CLL patients stratified by CD71/TFRC expression levels, based on publicly available proteome data from Herbst *et al*. Statistical analysis: Unpaired t-tests were used for comparisons between independent groups (Fig. S5a-k, and m). Kaplan–Meier survival analysis was applied for survival comparisons (Fig. S5n). Abbreviations: ‘n’ indicates the sample number; bars represent the mean; P value: *P < 0.05; **P < 0.01; ***P < 0.001.

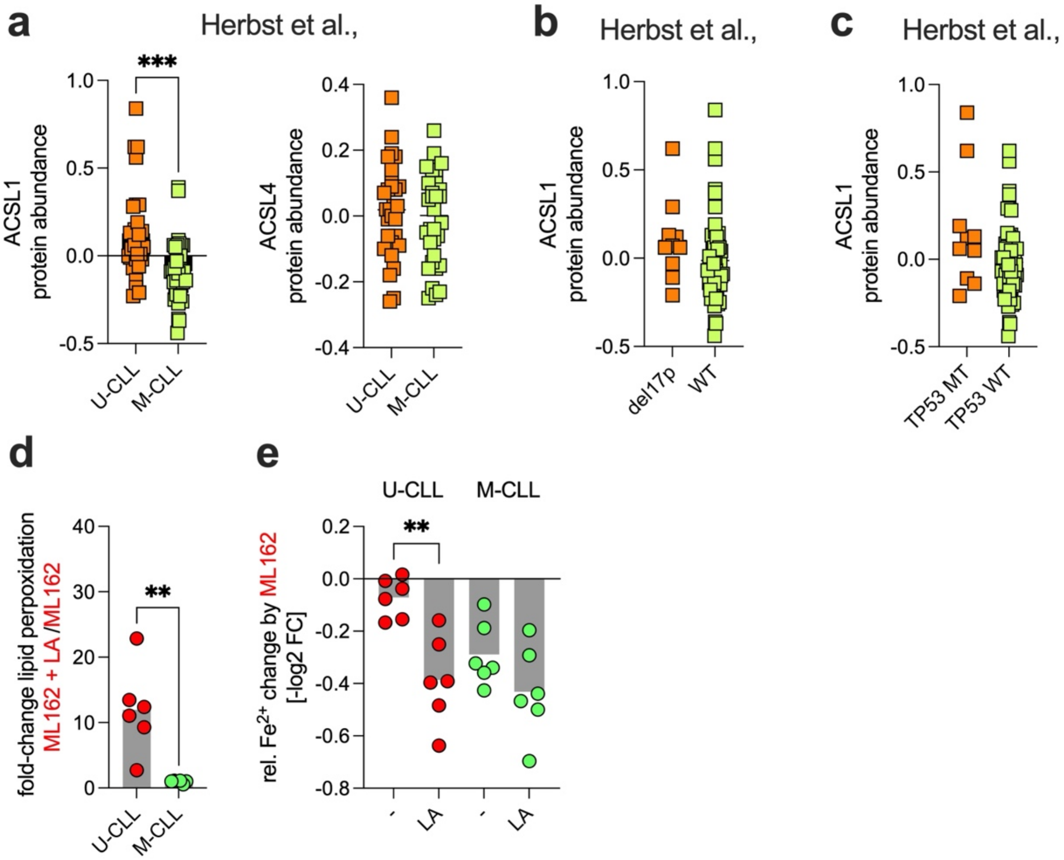

Supplementary Figure 6. Based on publicly available proteome data from Herbst SA *et al*. primary patient CLL samples were compared for their protein levels of **(a)** ACSL1 and ACSL4 between U-CLL (n=29) and M-CLL (n=35), and ACSL1 **(b)** between wildtype (WT) (n=53) and del17p (n=10), and **(c)** between TP53 mut (n=10) and TP53 WT (n=54). U-CLL (HG3, CII) and M-CLL (Mec-1, I83-E95) cells were cultured for 24 h in absence or presence of linoleic acid (LA, 10 µM) and then treated with 100 nM ML162. **(d)** The fold-change of lipid peroxidation in LA-pretreated compared to non-pretreated cells, as well as **(e)** the relative Fe^{2+} change in absence (-) and presence (LA) of linoleic acid triggered by 100 nM ML162 were assessed by FACS. Statistical analysis: Paired t-tests were applied for comparisons involving dependent (matched) samples (Fig. S6e) while unpaired t-tests were used for comparisons between independent groups (Fig. S6a-d). Abbreviations: 'n' indicates the sample number; bars represent the mean; P value: *P < 0.05; **P < 0.01; ***P < 0.001.

Supplementary Table 1. CLL patient characteristics.

UPN	age	sex	IGHV	TP53	del17p
12	62	m	M	NA	NA
17	69	m	M	NA	NA
18	60	m	M	NA	NA
22	67	m	M	NA	NA
29	64	f	M	NA	NA
37	52	m	U	NA	NA
57	65	m	U	NA	NA
63	71	f	M	NA	NA
77	71	f	U	NA	NA
93	69	f	M	NA	NA
97	57	f	M	NA	NA
102	55	m	M	NA	NA
122	62	m	M	NA	NA
134	79	m	M	NA	NA
135	43	m	NA	NA	NA
138	76	f	M	NA	NA
140	78	m	M	NA	NA
160	51	f	U	WT	NA
166	79	m	NA	NA	NA
169	71	f	U	NA	NA
172	34	m	M	WT	NA
173	48	m	M	NA	NA
175	76	m	M	NA	NA
176	89	f	NA	NA	NA
180	72	m	M	NA	NA
183	74	f	M	NA	NA
184	60	m	U	M	NA
185	72	m	U	NA	NA
186	70	m	NA	NA	NA
187	79	m	M	NA	NA
193	62	m	U	WT	NA
194	72	m	NA	NA	NA
196	58	m	M	NA	NA
197	51	m	M	NA	NA
202	74	m	M	NA	NA
208	76	f	M	M	Y
213	77	m	M	NA	NA
215	60	m	U	NA	NA
253	54	m	U	M	Y

256	79	f	U	WT	N
258	74	m	U	M	Y
259	67	f	U	WT	N
271	NA	m	U	WT	N
274	71	f	U	M	N
275	68	m	U	M	Y
276	80	f	U	M	N
277	66	m	U	WT	N
279	80	f	U	WT	N
283	79	m	M	WT	N
289	73	m	U	M	N
291	76	m	M	WT	N
294	71	f	M	WT	N
299	79	m	M	WT	N
300	66	f	U	M	N
309	75	m	U	WT	N
314	80	m	NA	NA	NA
315	69	f	U	WT	N
316	75	f	U	M	Y
318	73	f	M	WT	N
319	80	m	U	WT	N

Abbreviations: UPN, unique patient number; f, female; m, male, M, mutated; N, none; NA, not available; U, unmutated; WT, wild type; Y, yes.

Supplementary Table 2. Compound list.

Compounds	Concentration	Distributor
anti-CD71 (clone OKT-9)	2 µg/mL	Bio X Cell
Artemisinin	10 µM	MedChemExpress
L-Buthionine-(S,R)-sulfoximine	100 µM	Selleckchem
Deferoxamine mesylate	100 µM	Sigma-Aldrich
Erastin	10 – 160 µM	Selleckchem
Ferrostatin-1	25 µM	Sigma-Aldrich
Ibrutinib	10 µM	Selleckchem, MedChemExpress
9(Z),11(E),13(E)-Octadecatrienoic Acid	10 µM	Cayman Chemicals
ML-162	100 – 500 nM	Cayman Chemicals
N-acetyl-cysteine (NAC)	5 µM	Sigma Aldrich
Necrostatin-1	100 µM	Cayman Chemicals
QNZ (EVP4593)	1 µM	Selleckchem
(1S,3R)-RSL3	100 nM	Cayman Chemicals, MedChemExpress
Sorafenib	30 µM	Selleckchem
Triacsin C	5 µM	Cayman Chemicals
Venetoclax	1 – 50 nM	Selleckchem, MedChemExpress
z-YVAD-FMK	10 µM	MedChemExpress

Supplementary Table 3. Antibody list for multiparametric flow cytometry.

Antigen	Fluorochrome	Clone	Isotype	Identifier	Distributor
**anti-human**					
5-LOX	PE	polyclonal	Rabbit IgG	orb495075	biorbyt
ACSL1	FITC	polyclonal	Rabbit IgG	orb7204	biorbyt
AIFM2/FSP1	FITC	polyclonal	Rabbit IgG	orb686994	biorbyt
CD3	BV785	OKT3	Mouse IgG2a, κ	317330	Biolegend
	PE-Fire640	SK7	Mouse IgG1, κ	344860	Biolegend
CD5	FITC	UCHT2	Mouse IgG1, κ	300606	Biolegend
	PerCP	UCHT2	Mouse IgG1, κ	300618	Biolegend
CD19	APC-Fire810	HIB19	Mouse IgG1, κ	302272	Biolegend
	BV570	HIB19	Mouse IgG1, κ	302236	Biolegend
	PE-Cy7	HIB19	Mouse IgG1, κ	302216	Biolegend
CD20	APC-Fire810	S18015E	Mouse IgG2a, κ	375530	Biolegend
CD24	BV650	ML5	Mouse IgG2a, κ	563720	BD
CD27	BV711	M-T271	Mouse IgG1, κ	356430	Biolegend
	PE	O323	Mouse IgG1, κ	302807	Biolegend
CD36	BV650	CLB-IVC7	Mouse IgG1, κ	745324	BD
CD38	BV785	HIT2	Mouse IgG1, κ	303530	Biolegend
CD45	BV711	HI30	Mouse IgG1, κ	304050	Biolegend
CD71	BV786	M-A712	Mouse IgG2a, κ	563768	BD
	FITC	M-A712	Mouse IgG2a, κ	555536	BD
CXCR4	PE-Dazzle594	12G5	Mouse IgG2a, κ	306526	Biolegend
FTH1	PE-Cy7	polyclonal	Rabbit IgG	orb892191	biorbyt
FTL	CF405M	polyclonal	Rabbit IgG	orb395039	biorbyt
GCLC	AF532	OTI1A3	Mouse IgG1	NBP2-70830	bio-techne
GPx4	AF750	LHM2	Mouse IgG1, κ	NBP3-08253	bio-techne
IgD	BV421	IA6-2	Mouse IgG2a, κ	348226	Biolegend
LDL receptor	BUV496	C7	Mouse IgG2b, κ	750385	BD
LPL	Biotin	OTI3A10	Mouse IgG1	NBP2-71178	bio-techne
Nrf2	PE	EP1808Y	Rabbit IgG	ab223926	abcam
SLC11A2/DMT1	AF680	polyclonal	Rabbit IgG	bs-3577R	bioss
Streptavidin	BV605	NA	NA	405229	Biolegend
xCT/SLC7A11	CF647	polyclonal	Rabbit IgG	orb100617	biorbyt
**anti-mouse**					
CD3	BV510	17A2	Rat IgG2a, κ	100234	Biolegend
CD5	FITC	53-7.3	Rat IgG2a, κ	100606	Biolegend
	BV421	53-7.3	Rat IgG2a, κ	100618	Biolegend

CD19	PE-Cy7	6D5	Rat IgG2a, κ	115520	Biolegend
CD45	BV785	30-F11	Rat IgG2b, κ	103149	Biolegend
CD71	BV510	RI7112	Rat IgG2a, κ	113823	Biolegend

Abbreviations: NA, not applicable.

Supplementary Table 4. Fluorophores and dyes for multiparametric flow cytometry.

Dye/Compound	Parameter	Identifier	Distributor
7AAD	Late apoptosis, necrosis	420404	Biolegend
Annexin V (FITC or APC)	Early apoptosis	640941, 640945	Biolegend
BioTracker Cystine-FITC	Cystine uptake	SCT047	Merck
BioTracker Far-Red Fe^{2+}	Fe^{2+} levels	SCT037	Merck
BODIPY™ 581/591 C11	Lipid peroxidation	D3861	Thermo Fischer Scientific
CellROX™ Deep Red	Cellular ROS	C10422	Thermo Fischer Scientific
Ghost Dye™ Red 710	Viability	13-0871-T500	Tonbo
Ghost Dye™ Violet 510	Viability	13-0870-T500	Tonbo
MitoSOX™ Red	Mitochondrial ROS	M36008	Thermo Fischer Scientific
Phen Green™ SK	Fe^{2+} levels	P14313	Thermo Fischer Scientific
ThiolTracker™ Violet	Thiol groups	T10095	Thermo Fischer Scientific
Zombie NIR	Viability	423106	Biolegend

Supplementary Table 5. sgRNA for CRISPR-Cas9.

Target	sgRNA name	Target sequence	PAM
CD71/TFRC	Hs.Cas9.TFRC.1.AB	CTATACGCCACATAACCCCC	AGG
	Hs.Cas9.TFRC.1.AA	CAATATAAGCGACGTGCTGC	AGG

Abbreviations: sgRNA, single guided RNA; PAM, protospacer adjacent motif.
